# Lessons from the historical dynamics of environmental law enforcement in the Brazilian Amazon

**DOI:** 10.1038/s41598-024-52180-7

**Published:** 2024-01-21

**Authors:** Felipe S. M. Nunes, Britaldo S. Soares-Filho, Amanda R. Oliveira, Laura V. S. Veloso, Jair Schmitt, Richard Van der Hoff, Debora C. Assis, Rayane P. Costa, Jan Börner, Sonia M. C. Ribeiro, Raoni G. L. Rajão, Ubirajara de Oliveira, Marcelo Azevedo Costa

**Affiliations:** 1 Center for Remote Sensing (CSR), Federal University of Minas Gerais (UFMG), Av. Presidente Antônio Carlos, Belo Horizonte, MG 662731270-901 Brazil; 2 Laboratory of Environmental Services Management (LAGESA), Federal University of Minas Gerais (UFMG), Belo Horizonte, MG Brazil; 3 Brazil’s Institute of Environment and Natural Resources (IBAMA), Brasília, DF Brazil; 4 Center for Development Research (ZEF), University of Bonn, Bonn, Germany

**Keywords:** Environmental sciences, Ecology

## Abstract

Here, we analyze critical changes in environmental law enforcement in the Brazilian Amazon between 2000 and 2020. Based on a dataset of law enforcement indicators, we discuss how these changes explain recent Amazon deforestation dynamics. Our analysis also covers changes in the legal prosecution process and documents a militarization of enforcement between 2018 and 2022. From 2004 to 2018, 43.6 thousand land-use embargoes and 84.3 thousand fines were issued, targeting 3.3 million ha of land, and totaling USD 9.3 billion in penalties. Nevertheless, enforcement relaxed and became spatially more limited, signaling an increasing lack of commitment by the State to enforcing the law. The number of embargoes and asset confiscations dropped by 59% and 55% in 2019 and 2020, respectively. These changes were accompanied by a marked increase in enforcement expenditure, suggesting a massive efficiency loss. More importantly, the creation of so-called conciliation hearings and the centralization of legal processes in 2019 reduced the number of actual judgments and fines collected by 85% and decreased the ratio between lawsuits resulting in paid fines over filed ones from 17 to 5%. As Brazil gears up to crack-down on illegal deforestation once again, our assessment suggests urgent entry points for policy action.

## Introduction

Like many countries sheltering vast tropical forests, Brazil has sound environmental laws on paper, but their enforcement is often insufficient, especially in the Amazon. Still, Brazil stands apart, because it has historically developed unique institutional capacities that, in principle, allow for effective deforestation monitoring and law enforcement^1–3^. Variations in how this institutional setting was put to work have contributed to a sharp drop in deforestation by 84% between 2004 and 2012^4^, followed by a 60% increase^4^ until 2022. Since 2023, the newly elected government attempts to take back the reins on Amazon deforestation rates.

This historical dynamic makes the Brazilian Amazon an ideal laboratory to study interactions between deforestation and environmental law enforcement. Environmental law enforcement is an important inhibitor of tropical deforestation^5^. The reasons for variation in enforcement effectiveness and efficiency are poorly understood, however. Apart from political regime shifts and their impacts on governmental commitment^6–9^, institutional changes affecting environmental agencies and operational effectiveness are less visible but likely contribute to how law enforcement systems respond to the dynamics of deforestation^3^. Here, we compile and descriptively interpret new and comprehensive evidences for changes in how Brazilian institutions have enforced environmental law in the Amazon over the past two decades. Using a dataset of environmental law enforcement indicators, we document critical changes in the command-and-control regime for forest conservation in the Brazilian Amazon between 2000 and 2020. This allows us to identify changes in enforcement patterns that may have contributed to the rebound of deforestation rates since 2012 and extract lessons for effectively implementing Brazil’s new Plan for the Prevention and Control of Deforestation in the Amazon (PPCDAm), released in 2023.

### A short recent history of deforestation and public policies in the Brazilian Amazon

#### Early rise (1990–2004)

During the military dictatorship (1964–1985), Brazil’s government provided massive public support for infrastructure investments, such as roads^10,11^, to colonize the Amazon region. The replacement of forests by pastures and crops was endorsed as a means to accelerate economic growth and guarantee national security in the region^12^. As a result, numerous settlers migrated into the region^13^ and Brazil became a global provider of beef and soy^14–17^. Given limited law enforcement capacities at the time, deforestation skyrocketed in the 1990s.

Overtime, however, illegal deforestation and land grabbing became public policy issues. Political crises and presidential elections were often accompanied by increased deforestation^18^. Colonization projects were accompanied by frauds and land conflicts^19^, leading to violence and regional instability. Government plans aimed at combating deforestation were ineffective, and deforestation rates responded primarily to variations in the economic and political context rather than to environmental policy^18^. Annual forest loss fluctuated substantially since measurement began in 1988, with the highest annual rate in 1995 (29.1 thousand km^2^). While the 1995 peak was related to the June 1994 “Plano Real”, the rise from 2000 to the 2004 peak was closely associated with commodity prices, an effect that carried over during the initial decline after 2004^20,21^.

#### Command-and-control crackdown (2004–2012)

In 2004, when deforestation reached 27.8 thousand km^2^, a recently elected government by then launched the Plan for the Prevention and Control of Deforestation in the Amazon (PPCDAm)^22^. The Plan evolved through four phases, each one with specific priority actions—PPCDAm-I (2004–2008), II (2009–2011), III (2012–2015), and IV (2016–2020) (Supplementary Information: Figure S1). In 2023, after a three-year hiatus, the plan was relaunched.

In PPCDAm-I, efforts aimed essentially at enforcing existing laws and the designation of large tracts of public lands as protected areas^22^. Though criticized as “paper parks” in some parts of the world^23^, these protected areas acted as green barriers against deforestation based on research suggesting that formal protection discourages land grabbing^24^. In 2003, 86 federal conservation units (CUs) in the Amazon covered 36.4 Mha. By the end of PPCDAm-I in 2008, CUs coverage had almost doubled to 60.4 Mha (Supplementary Information: Figure S2). Between 2004 and 2008, 24 indigenous lands (ILs) added 8.06 Mha to help arrive at the current 106 Mha of protected areas (CUs + ILs), which conserve one-third of the remaining Amazon Forest in Brazil (Supplementary Information: Figure S3). In fact, both CUs and ILs were shown to have reduced deforestation rates under increasing external pressure^24–28^.

While dropping commodity prices played a role in reducing deforestation during the early years of PPCDAm-I^20^, command-and-control efforts also marked an “*the initial decline phase (2004–2007)*”. The actions taken during PPCDAm-I caused a significant drop in deforestation rates, from a peak of 27.8 thousand km^2^ in 2004 to 11.7 thousand km^2^ in 2007 (a 58% reduction)^3^. Yet, more effective enforcement measures and informative instruments followed suit. These included listing deforestation hotspots, barring bank loans to deforesters, and near-real-time satellite support for field operations^3,29^. Between 2008 and 2012 (*the effective command-and-control phase*), annual deforestation fell to 4.6 thousand km^2^ despite continued growth in agricultural production in the region. This reduction has been credited to a mix of public policies^1^, mainly law enforcement^2,3,5^, and, to a lesser extent, to the soy moratorium^30,31^ and agreements with the cattle sector^32^.

Amidst falling deforestation rates and international prices of export commodities, such as soy and beef^20,33,34^, the PPCDAm-II (2009–2011) gradually lost its centrality to the governmental agenda, shifting at the same time the focus toward land-tenure regularization of landholdings. Hence, conservation units expanded at a much lower rate over the second and third phases of the plan (Supplementary Information: Figure S2). Despite the focus on regularizing landholdings, monitoring and command-and-control actions continued to improve. Field enforcement operations (including asset confiscation and land-use embargoes, i.e., bans on productive uses of illegally deforested areas) focused on municipalities of so-called priority list—characterized by high rates of deforestation^33^. Priority municipalities were subject to administrative and economic disincentives, including restricted access to public credit schemes^35^. In addition, the federal environmental agencies streamlined internal administrative procedures and abandoned the centralized process of analyzing infraction notices. From 2009 onwards, notifications were analyzed in a decentralized manner by larger numbers of environmental analysts^36^ to hold offenders accountable more efficiently. Following all these measures, deforestation dropped to the lowest rate on record in 2012. In the same year, however, the Brazilian Forest Code (the legal basis for environmental law enforcement) was revised, granting amnesty to a large number of past offenders^37^. The law reform sent out a strong political signal suggesting laxer commitment of the government to conserve Amazon forests.

#### The return of deforestation (2013–2022)

Deforestation rates slowly began to rise throughout PPCDAm-III (2012–2015), which adopted a decentralized approach to share enforcement responsibilities with states and municipalities. It also placed even more emphasis on land tenure regularization and supported the CAR (a national online environmental rural registry), especially in the municipalities on the priority list^33^. As of 2022, CAR system contains 6.7 million records nationwide. Incentives to enter the CAR system are strong as it is a requirement for rural credit and formal land market transactions. Still, so far there is no evidence that land tenure or environmental registration would have deterred deforestation^38–40^.

In the last phase before its discontinuity in 2020, PPCDAm-IV (2016–2020) refocused on “economic and normative instruments”, including a potential forest offset market^41^ and payments for ecosystem services. However, Brazil’s Supreme Court questioned the offset mechanism and established a concept of “ecological equivalence”, which limited trading opportunities and effectively stalled the development of a market^42^. Instead, some progress was made concerning conservation payments, for example, with the approval of the Floresta + project by the Green Climate Fund. Moreover, the Amazon Fund had by 2020 contributed with USD 365 million^43^ to the PPCDAm. Roughly half of the assigned resources went to command-and-control actions, a quarter to promote sustainable rural development, and the rest went into land tenure regularization and research activities. Supporting sustainable rural development had been on the agenda since the beginning of the PPCDAm^44^, but received little attention^45^. Meanwhile, attempts to downsize and downgrade CUs (Supplementary Information: Table S1 and Figure S4) accompanied the comeback of higher deforestation rates.

After 2018, little or no progress was made toward the objectives of PPCDAm-IV, namely the designation and expansion of protected areas. In 2020, the government launched another national plan to control illegal deforestation and recover native vegetation^46^. Although comprehensive in its conception, it has not succeeded; deforestation continuously trended upward. In addition, the openly anti-environmental stance of Brazil’s government at that time encouraged illegal behavior^47^. In fact, the federal government supported by rural lobbies undertook several attempts to roll back past conservation achievements, namely presidential and legislative bills rewarding land grabbers^48^, permitting mining inside ILs^49^, and downsizing CUs (Supplementary Information: Table S1). The federal government also assigned political appointees (mostly military personnel) to chief positions at the environmental agencies (IBAMA and ICMbio), intending to undermine enforcement infrastructure, which the President called “the industry of fines”^50^. In 2021, Amazon deforestation had risen to 13 thousand km^2^, the highest level since 2007. During this period, forest fires devastated vast areas in the Amazon and the Pantanal regions and, in the Cerrado, deforestation reached 8.5 thousand km^2^ in 2021, the largest annual rate since 2016^4^. Similar patterns were observed in other Brazilian biomes^51^. In response to public critique at national and international levels, the government launched the operation “Verde Brasil” in 2019^52^. Led by the Brazilian armed forces, the program’s stated goal was to combat illegal deforestation and other environmental crimes in the Amazon. In addition, two more military operations took place in 2020 and 2021, i.e., “Verde Brasil 2” and “Samaúma”. The coordination of field operations was transferred from the environmental authorities to the Ministry of Defense and environmental enforcement agencies had to support their implementation by the Armed Forces. Deforestation rates continued to rise, nonetheless.

#### A come-back of law enforcement?

In 2023, the new government of President Lula da Silva reinstalled the PPCDAm. New efforts led by environmental agencies are underway to enforce the law, including special operations to extract illegal miners from indigenous lands, such as the Yanomami territory, where a humanitarian disaster was unfolding^53^. IBAMA´s infraction notices and sanctions in the Legal Amazon more than doubled compared to the average for the same period over the past four years, while deforestation alerts decreased by 43% compared to 2022 (Supplementary Information: Figure S5), showing a recovery of inspection capacity.

A fresh look at past achievements and setbacks in reducing deforestation can help inform the design of a new generation of conservation policies along with a more legal prosecution process to ensure deterrence and effective enforcement. To this end, we document how environmental law enforcement practices varied as the deforestation dynamics described above unfolded between 2000 and 2020. Specifically, we focus on infraction notices, asset confiscations, land-use embargoes, and completed judgments of environmental crimes. We also quantify changes in enforcement expenditures and operational efficiency in response to changes in administrative processes and the militarization of law enforcement in 2019 and 2020. This helps us illustrate how important it is to efficiently plan and carry out enforcement field operations.

## Results and discussion

Many counterfactual-based empirical studies have corroborated the causal role of changes in policy and enforcement practice in contributing to the sharp drop in Amazon deforestation rates between 2004 and 2012^54–57^. Most of these studies necessarily rely on reduced form of econometric specifications that yield average treatment effect estimates. Hypotheses on the underlying theoretical mechanisms are notoriously hard to test empirically. For example, infraction notices (i.e., the equivalent of a speeding ticket for traffic rule violation in forest law enforcement) have commonly served as a methodologically convenient indicator for enforcement activity in causal inference^54,57^. We must expect, however, that infraction notices become less effective in reducing deforestation, when legal authorities eventually fail to secure legal follow-up, for instance, via fine collection or asset confiscation. Thus, in lieu of a causal inference analysis, here we assess variations in a broad set of law enforcement indicators. The resulting lessons have implications for policy and can inform future empirical evaluation studies.

### Changes in enforcement intensity

**Figure 1:**
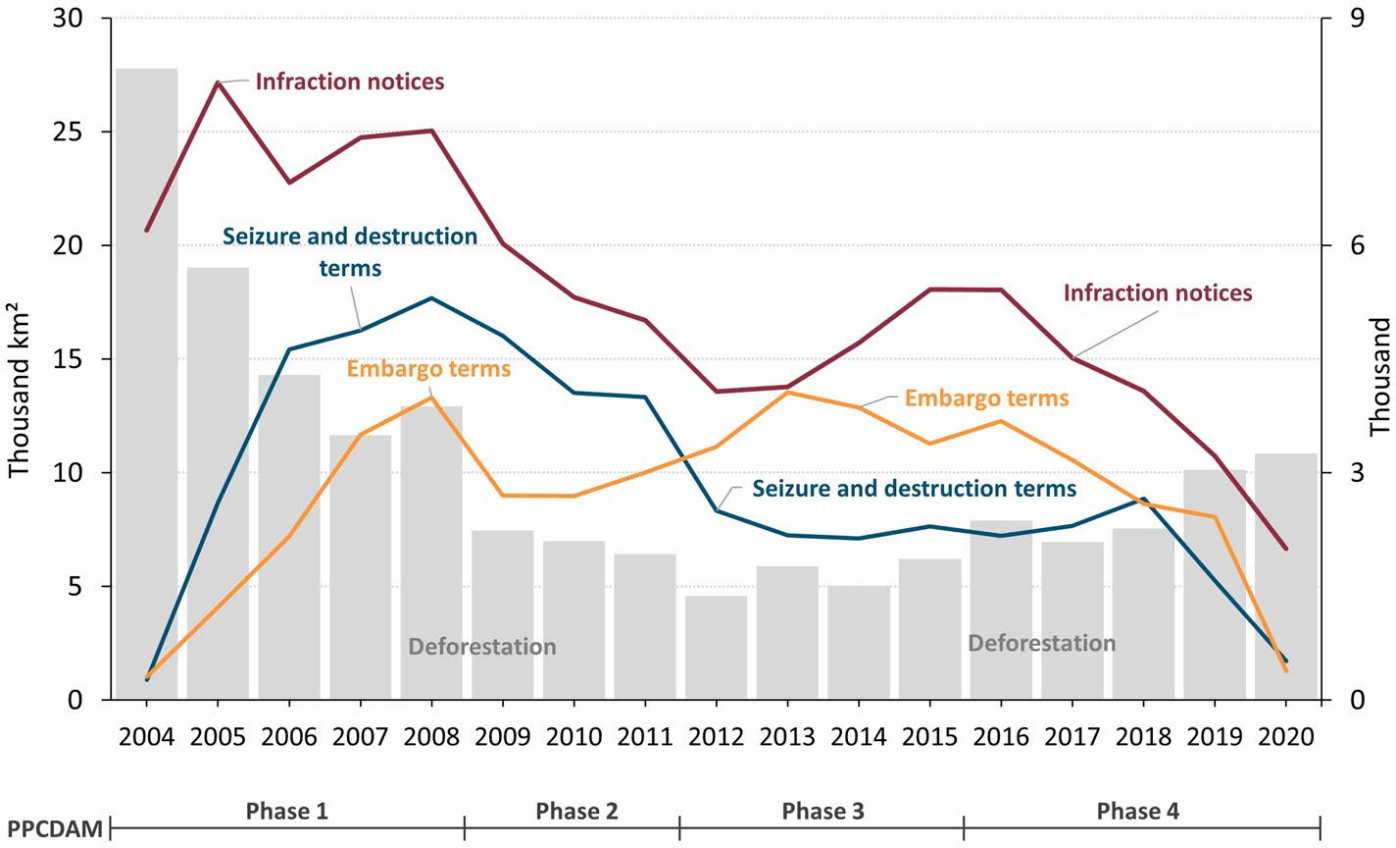
Number of infractions against the flora, asset confiscations and embargoes by IBAMA in the Legal Amazon over deforestation rates. Data source: deforestation^4^, inspection data^61^.

During PPCDAm I, the average annual number of “infraction notices linked to flora” (i.e., prospective fines for deforestation or other forms of native vegetation suppression) increased by 36% and the average fine value tripled (Supplementary Information: Figure S6). Meanwhile, the average annual deforestation rate dropped by 18% compared to the previous period (2000–2003). Coordinating enforcement efforts at the highest level of government (Chief of Staff) was essential to efficiently provide resources and political support for command-and-control actions^44^. This included, for example, increased funding, staffing, and training for IBAMA and ICMBio. At that time, many officials from these leading national institutions for environmental protection started attending graduate courses^22^, which improved technical and administrative capacities for planning and expanding field-based enforcement and legal prosecution. More resources were also provided to the National Institute for Space Research (INPE) to enhance its deforestation monitoring system for the Amazon (PRODES)^58^, which has informed official annual rates since 1988. Soon INPE made PRODES data publicly available and, in 2004, launched DETER, a monitoring system with near-real-time capability to detect large deforestation patches^59^. PRODES data were crucial to plan field inspection campaigns and served as an indicator for enforcement effectiveness^24,33,60^. In addition, DETER enabled rapid response operations, which reportedly enhanced the deterrence effect of field operations^29^. In the following years, several improvements were made to detect offenses and characterize environmental damages. This included DETER-Intenso, developed by INPE, which employs CBERS-4 WIFI imagery with 64 m pixel resolution and six revisits per month. While in 2004, only 5% of infraction notices had at least one geographic coordinate, in 2011, 61% of notifications were properly geolocated (Supplementary Information: Figure S6). Already in the second phase of PPCDAm (2009–2011), there was a 25% reduction in the annual number of infraction notices and the budget for field inspections was reduced in 2011 (Supplementary Information: Figures S6 and S7). Nevertheless, the drop in infraction notices was offset by greater operational efficiency as field inspections more often resulted in embargoes (Fig. 1). In both phases, over 19.6 thousand embargoes and 52 thousand fines, worth USD 2.6 billion and targeting 1.4 million ha, were issued alongside other sanctions aimed at decapitalizing offenders, such as asset confiscations and destruction of equipment used for illegal activities (Fig. 1).

More rigorous and efficient enforcement resulted in a backlash against the environmental policies, however^37^. Burdened by environmental sanctions, such as embargoes on productive areas of pasture and soy, rural interest groups successfully pushed for a reform of the Brazilian Forest Code (the legal basis for law enforcement actions) in 2012^37^. Alongside amnesty to 58% of all illegal deforestation before 2008, the new legislation compromised ongoing legal coercion processes. Using the number and values of fines for illegal deforestation between 1995 and 2008, we estimate that 11.4 thousand fines for infractions against the flora worth USD 1.07 billion were suspended due to the amnesty, thus contributing to a sense of impunity. In the following years of PPCDAm-III, the environmental agencies attempted to counteract the growing deforestation pressure. Between 2012 and 2018, 24 thousand embargoes and 32.3 thousand fines, worth USD 3.8 billion and targeting 1.9 million ha, were issued. This is equivalent to roughly 4 to 5 thousand infraction notices annually, but the average value of fines declined over time (Supplementary Information: Figure S6).

Brazil was hit by a political and economic crisis in 2015, which put additional pressure on the budget for environmental enforcement and forced IBAMA to turn to the Amazon Fund^43^ for resources in support of enforcement operations. The fund was suspended in 2019, however, after the Brazilian government violated the agreement by abolishing the civil society committees that oversaw the investments from the international donations (Norway and Germany). IBAMA’s annual inspection and law enforcement expenses fluctuated around USD 17 million during PPCDAm-I, increased to USD 20.3 million during the second phase, and reached USD 22.9 million in the third, with a peak at USD 30.9 million in 2013 (Supplementary Information: Figure S7). After a drop to USD 18.6 million in 2015, coinciding with the fiscal crisis, the expenditure partially recovered to USD 20 million, notwithstanding a drastic drop in infraction notices (Supplementary Information: Figure S7). From the point of view of public management, the decrease in the number of infraction notices, despite relatively stable budgets, implies growing inefficiency in public spending, namely via overspending on logistics and equipment. In addition, shrinking enforcement staff numbers hampered field operations. In 2010, IBAMA deployed 1,311 field inspectors, which were reduced to 965 by 2016 and only 691 by 2020. Until 2017, the inspection strategy focused on actions with strong potential deterrent effects (e.g., targeting large meatpackers and critical supply chain stakeholders). As the country’s political and economic crisis worsened, accompanied by changes in leading positions at environmental agencies, these operations gradually ceased and became more dispersed. The year 2018 marked a return to the traditional (pre-PPCDAm) inspection model, based on scattered and thus more costly field inspections followed by a gradual acceleration of deforestation rates in the years after.

**Figure 2:**
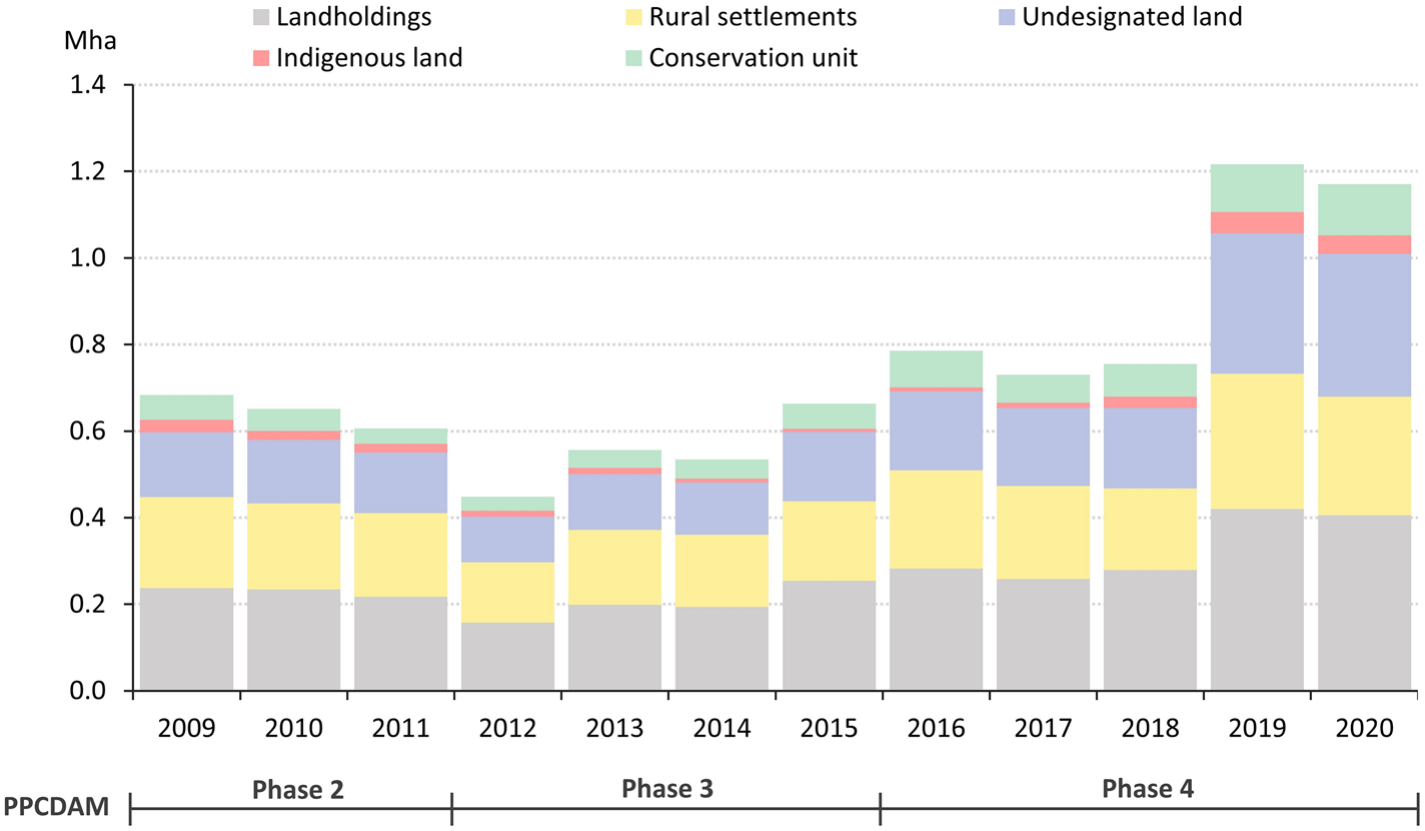
Annual deforestation per land category from 2009 to 2020 in the Amazon biome. Source: deforestation data from INPE^62^. Land category maps from maps.csr.ufmg.br.

Furthermore, political appointees without expertise in environmental law enforcement replaced many experienced leaders of enforcement operations. These factors arguably drove a significant setback in command-and-control effectiveness. IBAMA issued 4.6 thousand annual notices of infraction against the flora between 2012 and 2018 in the Amazon. In 2019–2020, only 2.6 thousand notices were issued yearly, a drop of 44%, despite a substantial rise in deforestation rates. The drop in infraction notices affected mainly the ten municipalities with the highest deforestation rates between 2018 and 2019, suggesting a paradigm shift away from federal commitment to forest conservation (Supplementary Information: Figure S8). This is in line with the accompanying cut-back in sanctions aimed at decapitalizing offenders, such as land embargoes and confiscation or destruction of physical assets. Compared to 2012–2018, the annual number of land embargoes and confiscations/destructions plummeted by 59% and 55%, respectively, in 2019 and 2020 (PPCDAM-IV) (Fig. 1 and Supplementary Information: Figure S9), while deforestation rates accelerated. The largest share of post-2008 deforestation is within landholdings (CAR records that do not belong to settlement projects or traditional communities’ lands), of which 90% is potentially illegal (Fig. 2). Yet, there was also a sharp increase in deforestation within undesignated lands, ILs, and CUs in 2019 and 2020, indicating a rise in land grabbing. Moreover, the CAR has become an instrument for land grabbing. We estimate that 11.3 thousand CAR records (1.4%), encompassing 2.9 Mha of land, overlap more than 25% with protected areas (ILs + CUs) in the Amazon and thus indicate illegal appropriation of public land.

### Changes in enforcement efficiency

**Figure 3:**
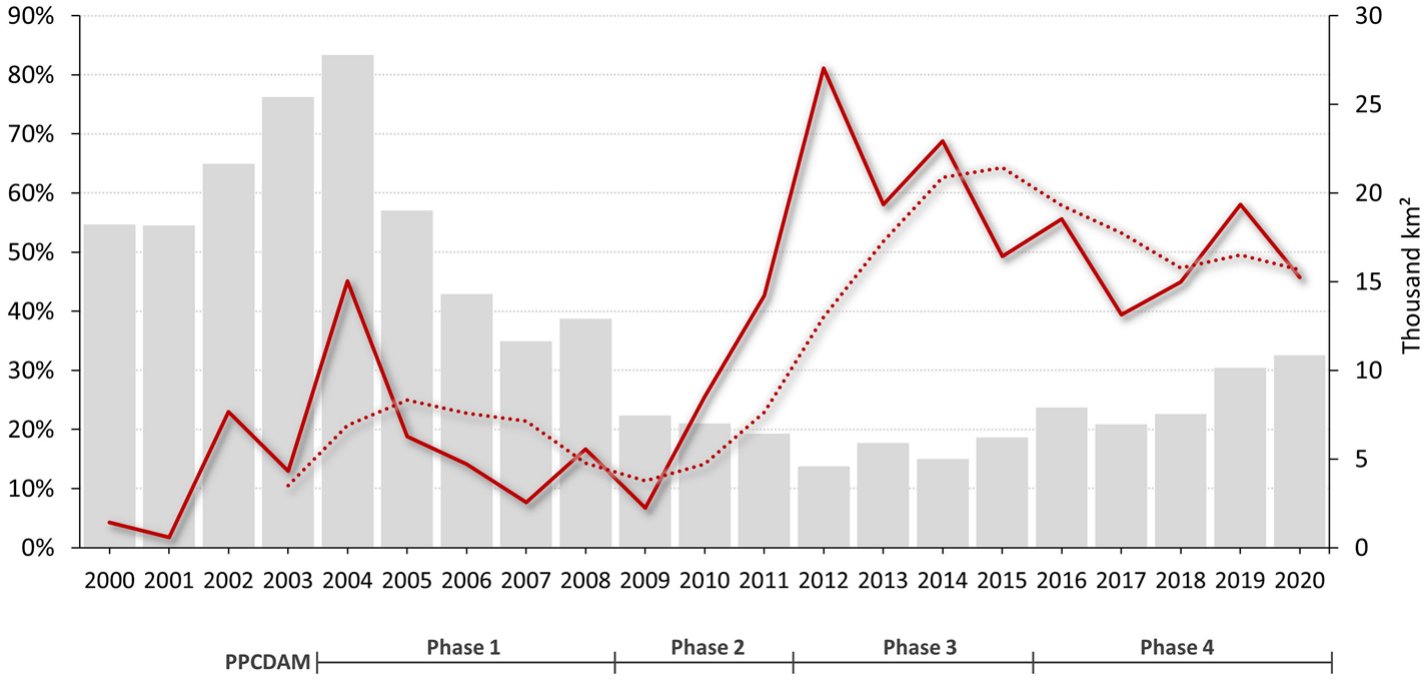
Pearson correlation between heat maps of annual fines and deforestation in the Amazon biome over annual deforestation rates. 4-year moving average in dashed line. Source: deforestation data from INPE^4^.

From 2000 to 2020, over 111 thousand infraction notices for crimes against the flora (over 99% resulting in prospective fines) were issued covering the Amazon in a geographically uneven way. Kernel (heat) maps show both the dynamics of deforestation and environmental law enforcement (Supplementary Information: Figure S10) and confirm the basic intuition that enforcement spatially correlates with illegal deforestation. We estimated Pearson correlation between the time-series maps of deforestation and fines as a proxy for the spatial targeting of IBAMA's field operations. The temporal trend indicates a growing spatial match from 2000 onwards, initially also influenced by the increasing number of infraction notices with geolocation information. This trend is indicative of improvements in deforestation monitoring leading to more informed operational planning among enforcement agencies. The spatial match rose steeply from 2009 peaking in 2012, when it coincided with the lowest annual deforestation on record (Fig. 3).

**Figure 4:**
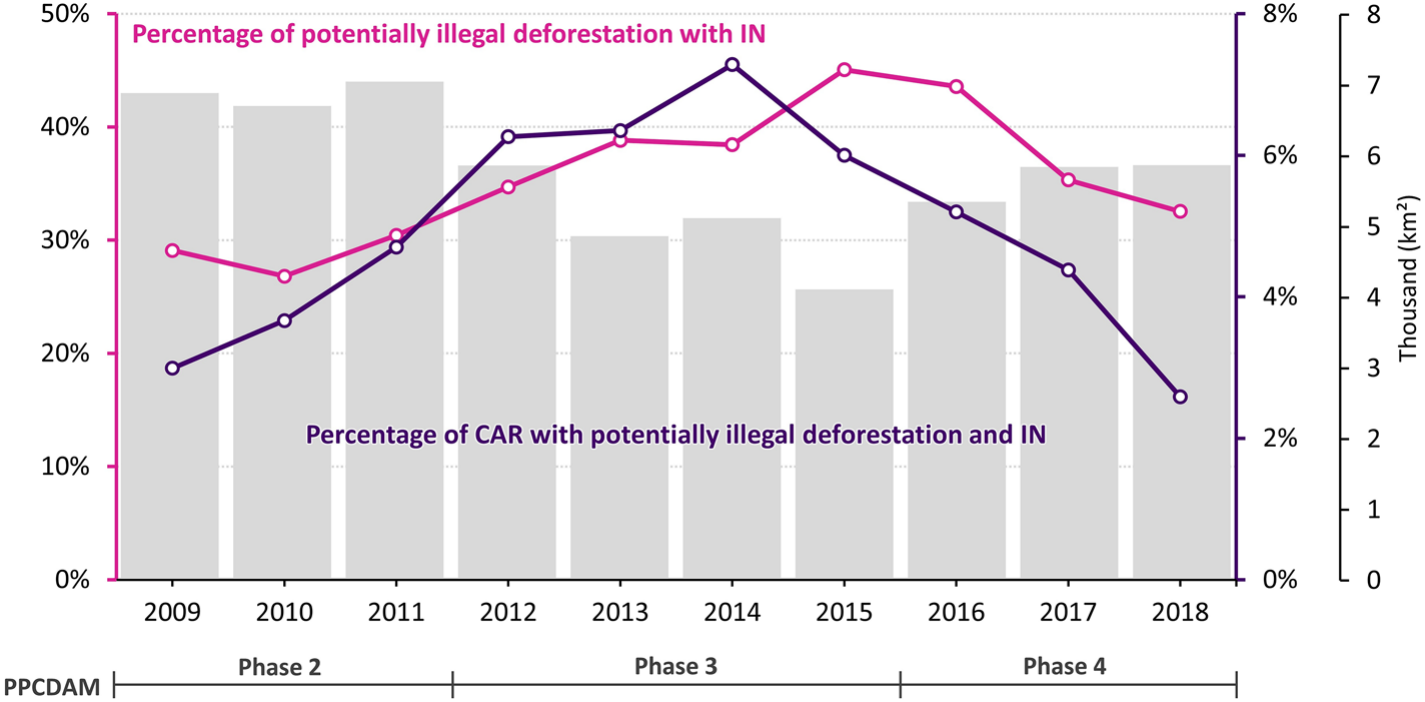
Annual areal percentage of potentially illegal deforestation with Infraction Notices (IN) and percentage of CARs of landholdings with Infraction Notices in the Amazon biome over annual deforestation rates. *Source*: deforestation data from INPE^4^.

Between 2009 and 2018, our analysis of potentially illegal deforestation shows that out of 362.6 thousand CAR-registered landholdings, 53.3 thousand deforested after 2008. In 43.9 thousand (82%) of these CARs deforestation was classified as potentially illegal (Supplementary Information: Figure S11). Only 10% of these CARs were formally notified and faced some kind of sanction. The annual percentage of CARs with infraction notices rose until 2014, when it reached 7.3%, and then declined to 2.6% by 2018 (Fig. 4). Although the percentage of CARs fined for illegal deforestation was quite low, field operations tended to target large patches of deforestation. Hence, in terms of area, the annual percentage of potentially illegal deforestation with infraction notices reached 45% in 2015 and then declined to 33% by 2018 (Fig. 4). Comparison of the number of landholdings and areas of illegal deforestation targeted by field operations suggest a drop in enforcement efficiency during and beyond the 2012–2014 period. Inefficiency increased even further after 2018 due to a drastic reduction in infraction notices and related sanctions (Fig. 1, Supplementary Information: Figures S12 and S13).

### Changes in the legal prosecution process

Environmental inspections aim to curb and prevent environmental damage by identifying and holding offenders accountable. Once an environmental violation is detected, environmental agencies must issue an infraction notice to inform citizens, companies, or institutions about the legal offense, the damage extent, and the monetary value of the fine. With the infraction notice, the environmental agency initiates the administrative process to investigate and punish offenders (Supplementary Information: Figure S14). The law enforcement procedure involves four phases: detection, inspection, judgment, and enforcement of sanctions, such as collecting fines or confiscations.

Operational budgets^63^ (enabling detection and field inspections) as well as sanctions for environmental crimes^64^ (legal coercion) co-determine the effectiveness of law enforcement^44^. Yet, deterrence also hinges on whether infraction notices effectively translate into legal prosecution, which depends on the efficiency of the legal administrative system^65^.

Although literature addresses the effectiveness of command-and-control and optimal enforcement strategies^66^, few studies have analyzed post-fine prosecution success^60^. Legal and institutional changes that hinder or excessively bureaucratize the legal process, such as the creation of a conciliation phase and the centralization of judgments in 2019^67^, increase the probability of impunity for illegal deforesters. The annual average number of fines paid between 2004 and 2018 dropped from 1071 to 480 in 2020, respectively (Supplementary Information: Figure S15). Two years later, roughly 250 conciliation hearings were concluded, representing less than 2% of the infraction notices issued in the same period^68^. As of 2021, IBAMA had nearly 99 thousand processes pending judgment, with 95 civil servants designated to act in the conciliation phase, 27 judging authorities at the first instance, and only one judging authority at the second and top instance, evidencing a human resource blackout to complete all judgment stages, which include appeals by offenders and second-instance decisions. Compared with 2012–2018, the average annual number of judgments (1st and 2nd instances) and the resulting fines paid plummeted by 97% and 85%, respectively (Supplementary Information: Figures S15 and S16). The number of paid fines had also fallen before, but the ratio between lawsuits resulting in paid fines over filed ones, which was already low, dropped from 17 to 5% after 2018 (Supplementary Information: Figure S15). Taken together, these changes have weakened the law enforcement system by reducing the likelihood that environmental crimes are effectively punished.

### Changes in institutional capacity and militarization

At the federal level, IBAMA and ICMBio are the agencies responsible for environmental law enforcement. They can request support from other federal and state government bodies, including security forces, such as the army and the federal police. Due to the historically low operational capacity of state-level environmental agencies, IBAMA and ICMBio have taken a prominent role controlling environmental crimes in the Amazon. For example, IBAMA and ICMBio accounted for 75% of embargoes issued in Pará and Mato Grosso before 2019. These two states accounted for roughly 53% of the total deforestation in the Amazon between 2011 and 2020^4^ (Supplementary Information: Figure S17). Even when state-level enforcement efforts increased to fill in the gap left by the pullback of the federal agencies, general accountability remained low. In 2020, only 13% and 24% of landholdings with deforestation in Pará and Mato Grosso were noticed or sanctioned by both state and federal agencies (Supplementary: Table S2).

In 2019, the leading role of IBAMA and ICMBio in environmental law enforcement was transferred to the Brazilian Armed Forces^52^. Despite the massive use of resources by the military, there was no significant reduction in deforestation rates, nor in wildfires (Fig. 3, Supplementary Information: Figure S18). Deforestation alerts increased by 113% and 60% during the military-run operation “Verde Brasil” in 2019^52^ and the subsequent “Verde Brasil 2”^69^ (Supplementary Information: Table S3). Also, the extent of burned areas in 2020 was on a par with that of 2010 (Supplementary Information: Figure S18), a year of extreme drought^70^. Nonetheless, inspection operations were far below the historical average, especially in 2020 (Fig. 1). In comparison within the 2004–2018 period, infraction notices for crimes against the flora fell by 65% in 2020. Additionally, confiscations and destructions of equipment and embargoes were down by approximately 83% and 87%, respectively (Fig. 1). And, while average annual expenditures more than tripled during 2019 and 2020 (USD 21.1 million to USD 67.4 million), deforestation rates increased by 62% compared to the annual average between 2009 and 2018 (Figs. 1 and 4).

**Figure 5:**
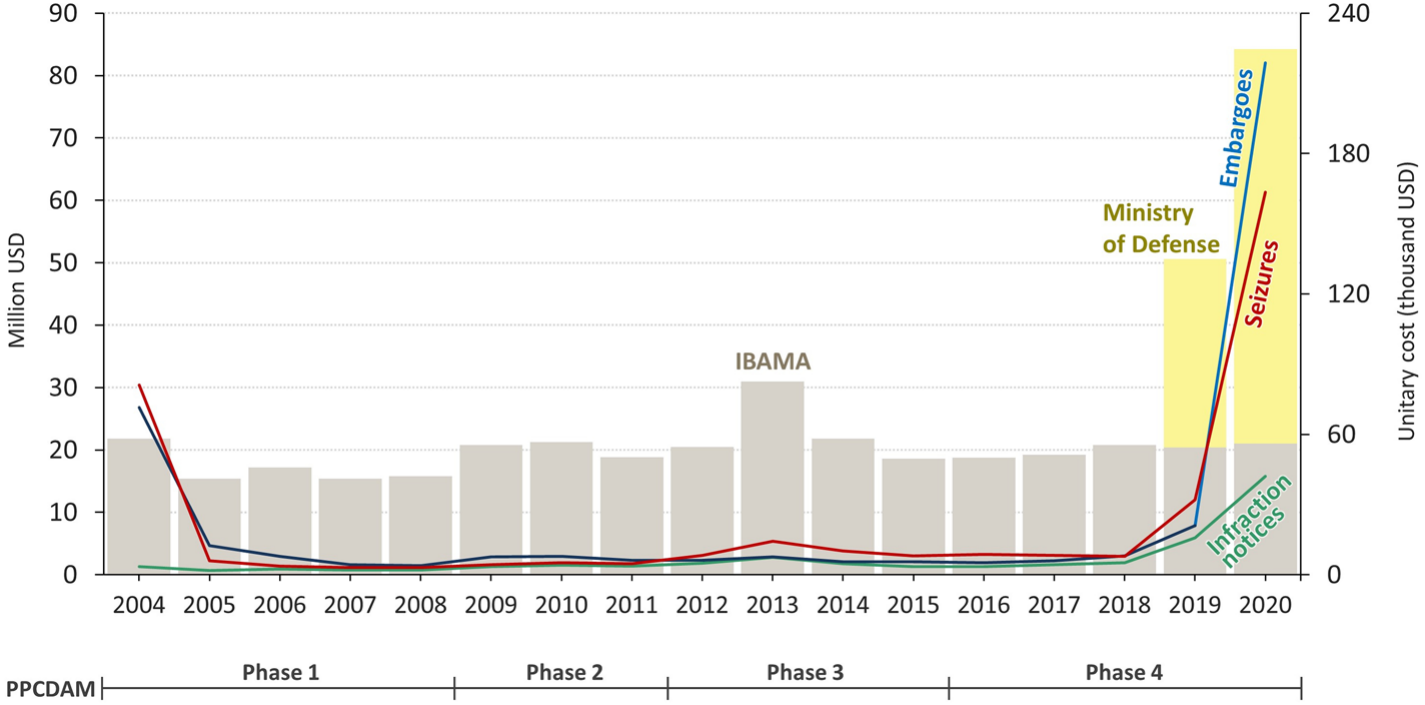
Expenditures of IBAMA and the Ministry of Defense for environmental enforcement in the Legal Amazon. *Source*: Brazilian Open Data Portal and data obtained through the Law on Access to Information (available at https://dados.gov.br)^61^.

From 2004 to 2020, IBAMA's total spending on inspection in Brazil amounted to USD 338 million (Fig. 5). The costs of the military operations in 2019–2020 (Verde Brasil I and II) totaled USD 90.3 million, which equals to about a third of the pre-militarization period (Fig. 5). All this implies a massive drop in the operational efficiency of law enforcement under the military leadership. Between 2004 and 2018, the average cost of issuing an infraction notice was USD 3.8 thousand. In 2019 and 2020, this value amounted to USD 15.7 thousand and USD 42.1 thousand, respectively. For embargoes, the unitary cost went from USD 11.1 thousand between 2004 and 2018 to USD 119.9 thousand in 2019 and 2020. Considering IBAMA’s historical average expenditure per infraction notice, its performance could presumably triple as a function of resources spent only on the operation “Verde Brasil 2” (from May to December) (Fig. 5).

Undeniably, the Armed Forces can play an important role in offering security and logistic support to IBAMA and ICMBio inspectors. However, our results indicate that the shift in operational leadership from specialized environmental agencies towards military personnel not only failed to achieve its objectives, but also wasted scarce public funds.

## Conclusion

The causal role of environmental law enforcement in reducing Amazon deforestation has been well-documented in the literature. For example, Hargrave and Kis-Katos^54^ estimated that a 1% increase in fine intensity (number of fines divided by the area of forest in a municipality) reduces deforestation by 0.2%. Börner et al.^60^ found that an additional fine reduces deforestation by 3.965 (± 1.977) and 9.868 (± 5.304) ha in the subsequent year, for the states of Mato Grosso and Pará, respectively, and Assunção et al.^34^ estimated that deforestation observed from 2007 through 2011 was 75% smaller than it would have been in the absence of fines^57^. Nevertheless, environmental law enforcement takes place under a complex policy mix and interacts with market dynamics^34^. The law and economics literature prescribes smart instrument mixes to solve environmental problems during fiscal austerity, arguing that command-and-control policies can encourage investments in environmentally friendly production practices^66^. Private and voluntary sustainability commitments may complement state regulation, but can hardly replace it^31^. Yet the results presented here indicate that law enforcement remains the backbone of any effective policy mix aimed at forest conservation^56,71,72^.

Comparing the unit cost of military-led field operations with those of IBAMA-led field activity, we found that the militarization of field operations significantly raised enforcement costs without tangible effects on deforestation. The efficiency-loss in public spending on environmental inspection stresses the importance of an enforcement strategy informed and supported by science and technology. This must include the use of DETER-intenso and the CAR to quickly identify offenders and be accompanied by effectively targeted field operations to issue fines and embargoes followed by expeditious legal prosecution.

Brazil’s institutional framework for fighting illegal deforestation and other environmental crimes used to be considered as one of the most advanced and comprehensive in the world^73^. This study has documented several entry points for governance interventions needed to reestablish this reputation. Merely increasing infraction notices and embargoes may be insufficient to reduce deforestation, however. Fines and sanctions that exist purely on paper cannot reduce deforestation and effectively waste substantial public resources spent on expensive field operations. Our analysis illustrates that public expenditure must be matched with good governance to protect the world’s largest tropical rainforest effectively. To this end, there is a need to constitute task forces to expedite the judgments of fines as they may expire after five years without administrative action.

Moreover, illegal deforesters have learned how to bypass controls and continue to sell their produce through intermediates that hide their origin^31^. Also, financial institutions and sanitary agencies are not doing enough to clear supply chains, as most deforesters can still obtain loans and cattle movement authorizations^74^. In that context, the implementation at the national level of public instruments able to expose deforestation from direct and indirect commodities suppliers, such as the SeloVerde platform in Pará (https://www.semas.pa.gov.br/seloverde) and Minas Gerais (https://seloverde.meioambiente.mg.gov.br), jointly with large-scale environmental regularization programs, will be crucial to reduce deforestation in the years to come. Official Data publicly available on environmental compliance at the farm level and supply chain transparency can also help discourage counterattacks by rural lobbies aimed to relax environmental laws again. In sum, Brazil has the right tools and recently signaled a renewed political will to fight deforestation. As such, both federal and state-level governments are likely to enjoy substantial international support in the endeavor to reinstall environmental rule of law in the Amazon region and beyond. A significant challenge lies ahead, nonetheless, because both the legal and the enforcement system must rebuild credibility also domestically.

## Methods

### Changes in enforcement intensity and in the legal prosecution process

We developed a comprehensive database for the Amazon encompassing all available records of infractions notices, prospective fines, embargoes, equipment confiscation/destruction, and administrative judgments between 2000 and 2020 (Supplementary Information: Table S4). IBAMA inspection data between 2000 and 2020 were obtained from the Brazilian Open Data Portal (https://dados.gov.br/dataset?q=ibama, downloaded on 03/17/21, 06/06/21, 10/03/22 and 10/03/23) and the Information Access Platform (https://www.gov.br/acessoainformacao/pt-br)^61^. We removed duplicate records and filtered data for the states of the Legal Amazon: Amapá, Acre, Amazonas, Pará, Rondônia, Roraima, Tocantins, Mato Grosso and Maranhão. We used only infraction notices and fines related to crimes against the flora^75^. We also accounted for all confiscations/destructions and embargoes since it was impossible to identify a unique key and separate the deforestation sanctions from other crimes against the flora. Enforcement data for the states of Mato Grosso and Pará were obtained from the geodata website of the State Secretariat of Environment—SEMA/MT^76,77^ and Pará’s official list of illegal deforestation together with infraction notices from SEMAS/PA’s database^78,79^. Our overall analyses employ only federal data (i.e., IBAMA’s) covering all the states of the Legal Amazon. For Mato Grosso and Pará we were able to include the available state-level data.

We further analyzed annual deforestation from 2009 to 2020 in the Amazon biome per land category, including landholdings on the CAR, rural settlements, undesignated land, conservation units and indigenous lands. CAR boundaries come from Pinto et al.^80^ and the other spatial data are listed in Supplementary Information: Table S4.

### Changes in enforcement efficiency

We derived kernel maps (bandwidth = 30 km) for annual deforestation from PRODES data and annual infraction notices with geolocation, which were rasterized to 30 m spatial resolution to fit the PRODES data. Kernel density estimates a continuous probability density (pdf) function over space based on a set of discrete sample event locations and a defined bandwidth—a parameter that controls for the smoothness of the pdf^81,82^. These maps are also known as heatmaps because the stronger the red color, the more concentrated the event (Supplementary Information: Figure S10). We then estimated Pearson correlations, pixel by pixel, to measure the spatial match between infraction notices and deforestation on an annual basis. Because we want to assess the spatial match between the concentration of fines and hotspots of deforestation, we set the lowest quartile of both deforestation and fine kernel maps to null values before the correlation analysis (Supplementary Information: Figure S10). Correlation coefficients vary between − 1 and 1, where 1 represents a perfect match. Spatial analysis was performed using Dinamica EGO^83^.

We only consider infraction notices applied after illegal deforestation took place to ensure that notifications are indeed related to deforestation (77% of the total landholdings with infraction notices). To do so, we laid the geolocation of infraction notices over our annual maps of potentially illegal deforestation between 2009 and 2018 per landholding on the CAR system^31^. We only account for deforestation larger than 6.25 ha within a landholding, as this size corresponds to the spatial resolution of PRODES^62^. Potentially illegal deforestation within a landholding is characterized as either occurring on Riparian Preservation Areas or in the presence of a Forest Code (FC) deficit (native vegetation area below law requirements) or insufficient Legal Reserve area, e.g., forest remnants occupying less than 80% of the landholding area in the Legal Amazon region.

We consider all embargo records (affidavits) due to the uncertainties in overlapping embargoed areas (polygons) with infraction notices and CAR boundaries. Yet, we estimate that 80–90% of all embargoes in the Amazon are related to crimes against the flora by cross-referencing infraction notices and embargo records.

Our big geodata analysis of the FC balance and potentially illegal deforestation utilizes the methodology developed by^31^ and applied to develop the Selo Verde and X-Ray of the CAR platforms (https://www.semas.pa.gov.br/seloverde/, https://csr.ufmg.br/radiografia_do_car/). To calculate the FC balance for the 362.6 thousand landholdings, we integrated their boundaries from the CAR to land-use maps from Mapbiomas^84^, annual deforestation maps from PRODES^62^ and river streams and water bodies from ANA^85^. This required the development of a set of geoprocessing algorithms employing PostgreSQL and PostGIS extension and Dinamica EGO freeware^83^. Dinamica EGO retrieves the CAR vector data from PostGIS to perform the FC calculation, employing the aforementioned set of maps as input, through parallel processing pipelines for lots of CARs, in which the amount of records depends on the number of available processors. The FC results per landholding are then integrated with annual deforestation maps^62^ to calculate whether each deforestation patch within a specific landholding is potentially illegal or not. All other spatial analyses are also performed using Dinamica EGO.

### Changes in institutional capacity and militarization

Given the main objective of operations Verde Brasil (illegal deforestation and fires), we filtered infraction notices issued and fines only for crimes against the flora.

The analysis for the operational efficiency of the pre-and military operations was calculated using the ratio between government expenditures and the results (sanctions) of inspections, as follows:

$${OE}_{i,y}=\frac{{E}_{y}}{{I}_{i,y}}$$

where, *OE*_*i,y*_ is the operational efficiency for a type of sanction *i* (i.e., fines, confiscation and confiscation/destruction or embargoes) in a year *y*, *Ey* is the total expenditure in a year *y* and *I*_*i,y*_ is the number of sanctions *i* issued in year *y*. As fines, embargoes, and confiscation/destruction generally come from the same infraction notice, the unit costs are not equivalent to the sum.

To calculate the government costs, we accounted for the net expenses of IBAMA and the Ministry of Defense assigned to environmental inspections in the Legal Amazon between 2004 and 2020. We selected budget actions called “21BT” and “218X” for the Ministry of Defense. 21BT is a budget item created exclusively for the Verde Brasil operation, whose data were obtained from the Integrated Planning and Budget System (SIOP)^86^. 218X, however, is a much broader item that covers other military operations, so the amount destined only for Verde Brasil was obtained directly from the Ministry of Defense through the Access to Information Law. Almost half of military expenses were allocated to consumable materials, e.g., aviation supply and fuel, 39% on other parties’ services (e.g., technicians, telecommunication services, maneuvering and patrolling services) and 13% to purchase equipment and permanent material (e.g., vehicles). The remainder was used mainly for per diem and transportation costs.

For IBAMA’s budgetary items, we accounted for items 2943, 2944, 2945, 2946, 2947, 6037, 6124, 6307, 6309, 6485, 8515, 20WE, 214N, and 21BS, whose data were also obtained from the SIOP Portal. As measuring the exact amount allocated to the states in the Legal Amazon was impossible, we utilized the entire budget as a proxy. All monetary values were adjusted using the IPCA (Ample Prices to the Consumers) index and converted at R$ 5 per USD 1^87^.

### Supplementary Information

Supplementary Information.

## Data Availability

The datasets used and/or analysed during this study are available by the corresponding author upon reasonable request.
