# Supplementary material for: Lessons from the historical dynamics of environmental law enforcement in the Brazilian Amazon
Source: Sci Rep. 2024 Jan 21;14:1828. doi: 10.1038/s41598-024-52180-7 (PMC10800348; doi:10.1038/s41598-024-52180-7)
Supplement: Supplementary file 1 — Supplementary Information. [file 41598_2024_52180_MOESM1_ESM.pdf]

Supplementary Information

Lessons from the historical dynamics of environmental law enforcement in the Brazilian Amazon

Felipe S. M. Nunes[1*], Britaldo S. Soares-Filho[1], Amanda R. Oliveira[1], Laura V. S. Veloso[2], Jair Schmitt[3], Richard Van der Hoff[2], Debora C. Assis[1,2], Rayane P. Costa[2], Jan Börner[4], Sonia M. C. Ribeiro[1], Raoni G. L. Rajão[1,2], Ubirajara de Oliveira[1], Marcelo Azevedo Costa[1].

[1]Center for Remote Sensing (CSR), Federal University of Minas Gerais (UFMG), Belo Horizonte, MG, Brazil.

[2]Laboratory of Environmental Services Management (LAGESA), Federal University of Minas Gerais (UFMG), Belo Horizonte, MG, Brazil.

[3]Brazil's Institute of Environment and Natural Resources (IBAMA), Brasília, DF, Brazil

[4]Center for Development Research (ZEF), University of Bonn, Bonn, Germany.

*Corresponding author at: Av. Presidente Antônio Carlos, 6627, Belo Horizonte, Brazil, 31270-901.
Email address: felipesm.nunes@gmail.com (Felipe S. M. Nunes).

This file includes:

Figures S1 to S18
Tables S1 to S4
SI References

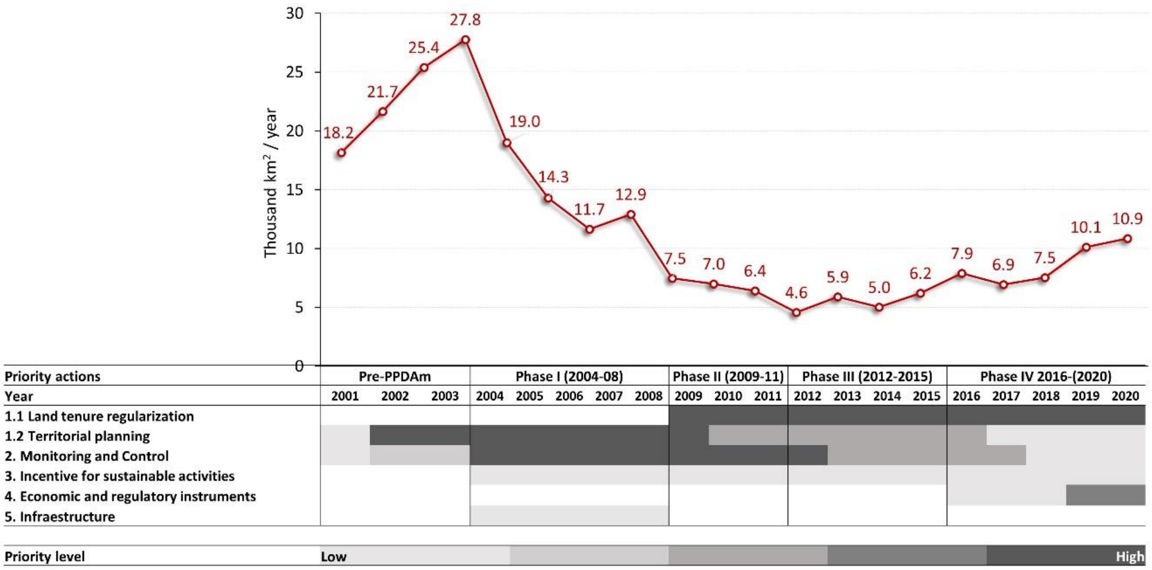

Fig. S1. Phases of the Action Plan for the Prevention and Control of Deforestation in the Legal Amazon (PPCDAm) and its priority actions (i.e., dark gray) along with annual rates of deforestation in the Amazon from Prodes/INPE.

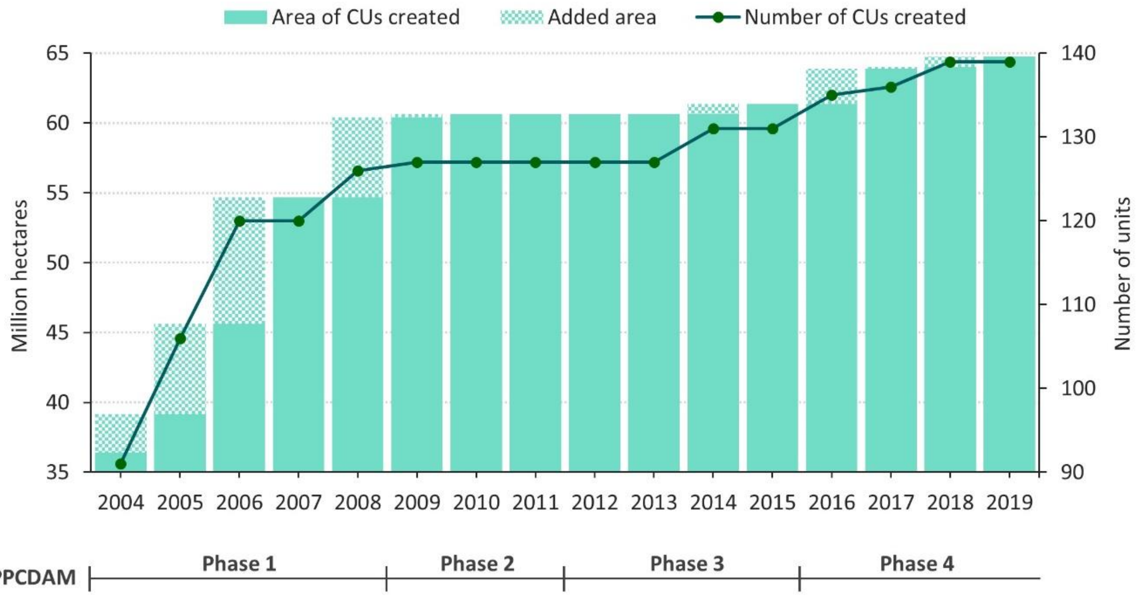

Fig. S2. Numbers and new areas of federal Conservation Units (CUs) created in the Legal Amazon from 2004 to 2019.

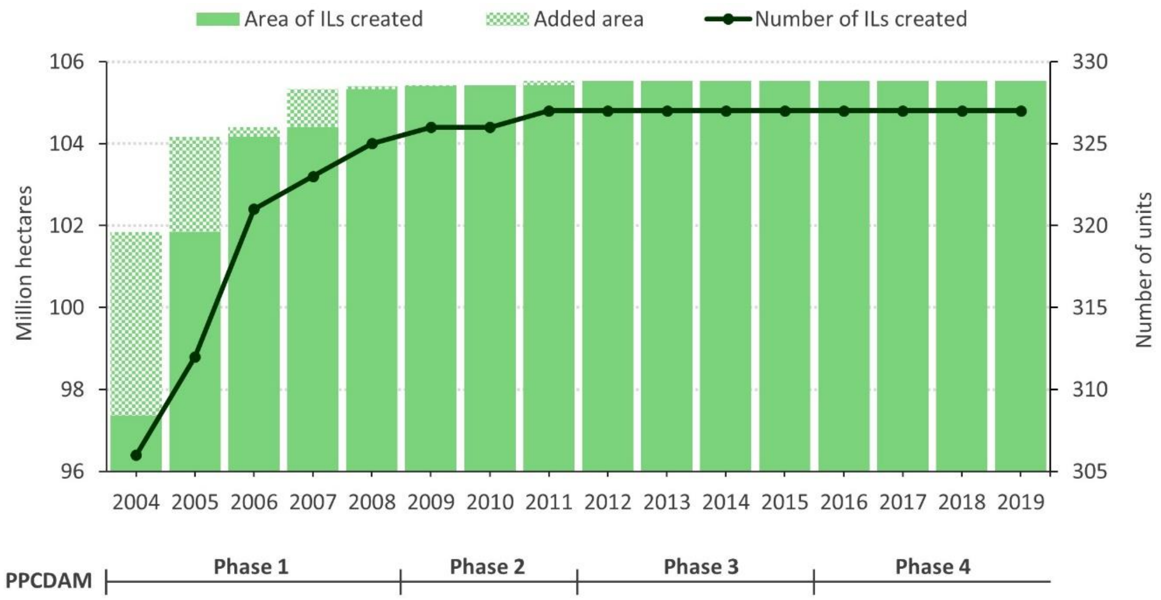

Fig. S3. Numbers and new areas of homologated indigenous lands (ILs) in the Legal Amazon from 2004 to 2019.

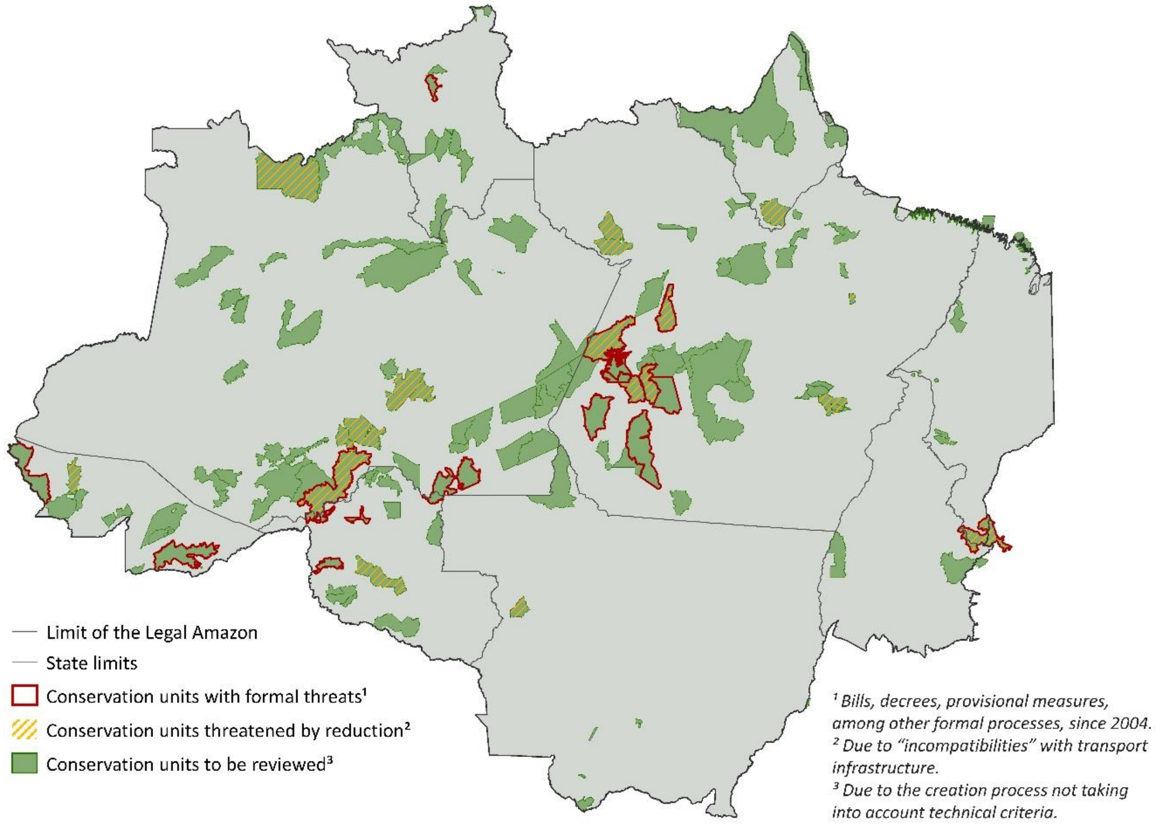

Fig. S4. Conservation Units at risk or effectively downsized, downgraded or degazetted in the Legal Amazon. Source: MMA (2021). Figure generated by ArcGIS Pro (https://www.esri.com/pt-br/arcgis/products/arcgis-pro/overview).

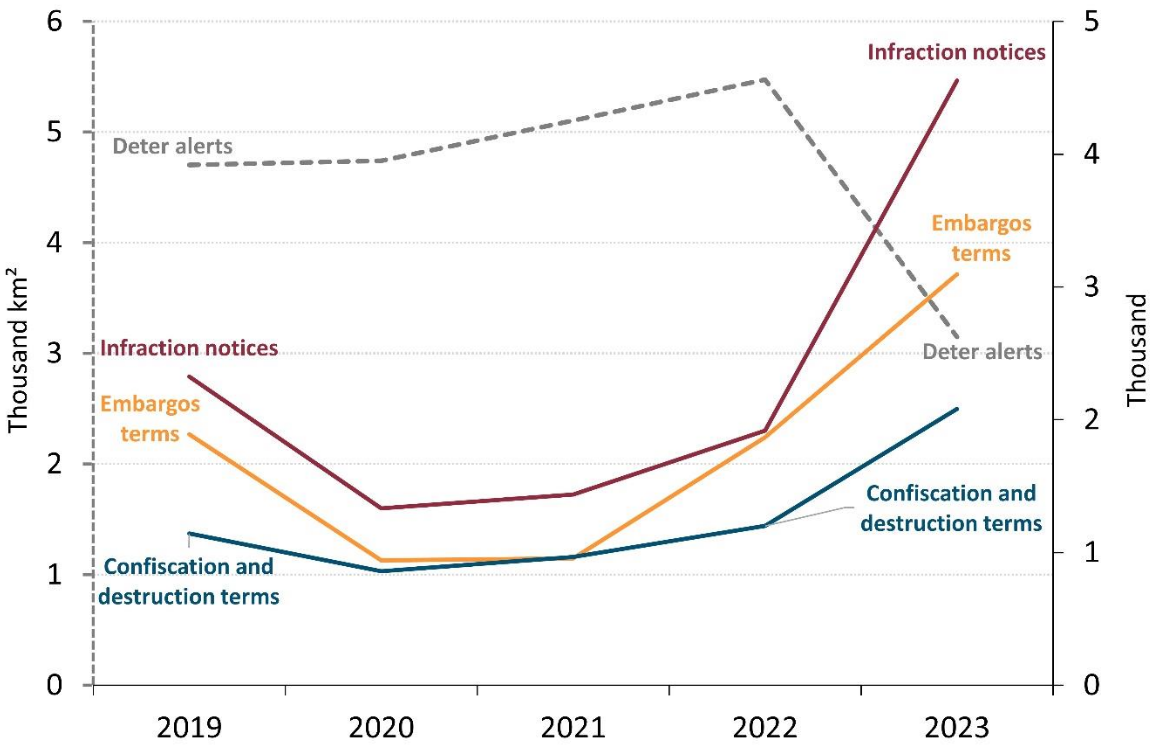

Fig S5: Number of infractions against the flora, asset confiscations and embargoes by IBAMA in the Legal Amazon over deforestation alerts from January to August. Sources: INPE (2023) and IBAMA (2023).

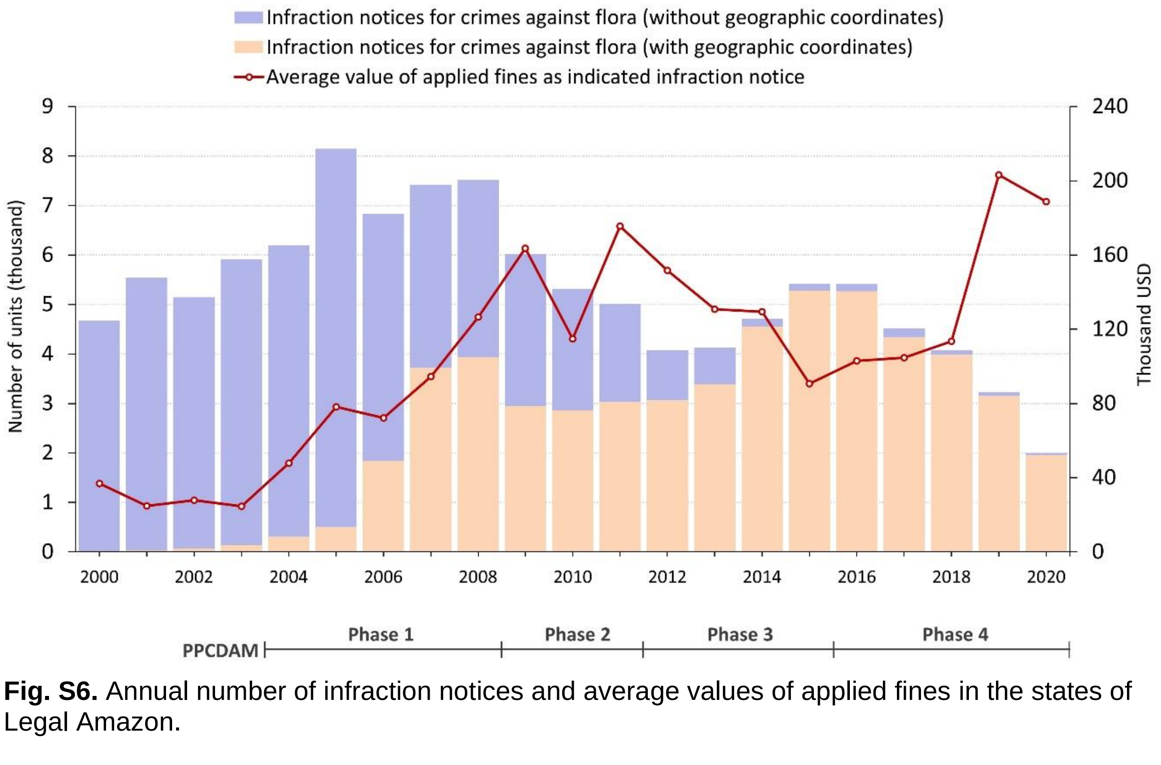

Fig. S6. Annual number of infraction notices and average values of applied fines in the states of Legal Amazon.

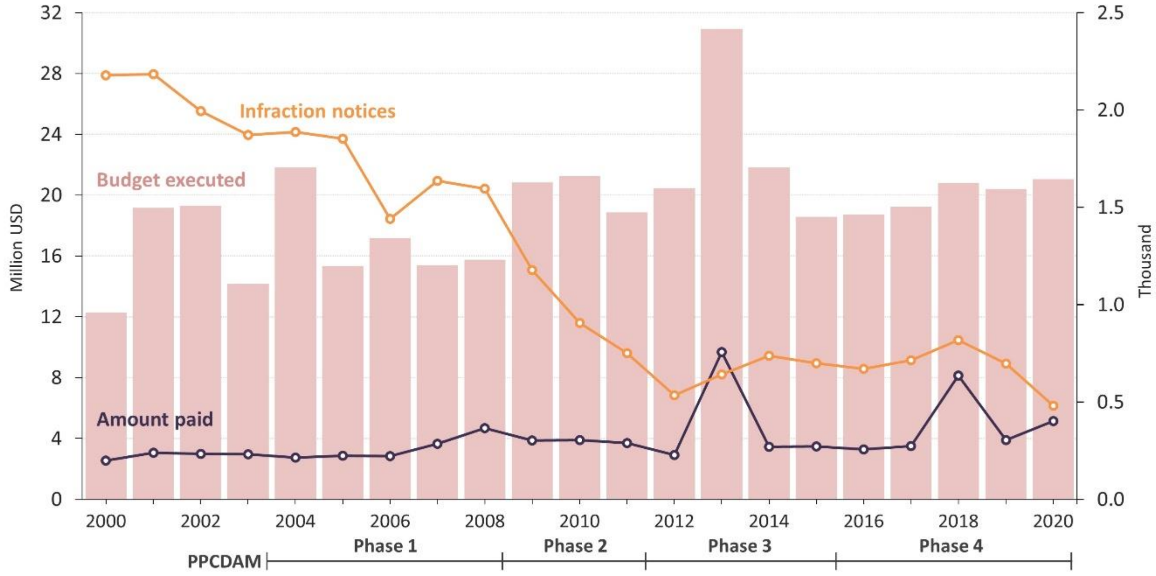

Fig. S7. IBAMA´s infraction notices issued, fines paid, and expenditures for environmental inspection. Values were adjusted for inflation and converted to USD using a rate of R$ 5 per U$ 1 (BCB, 2021).

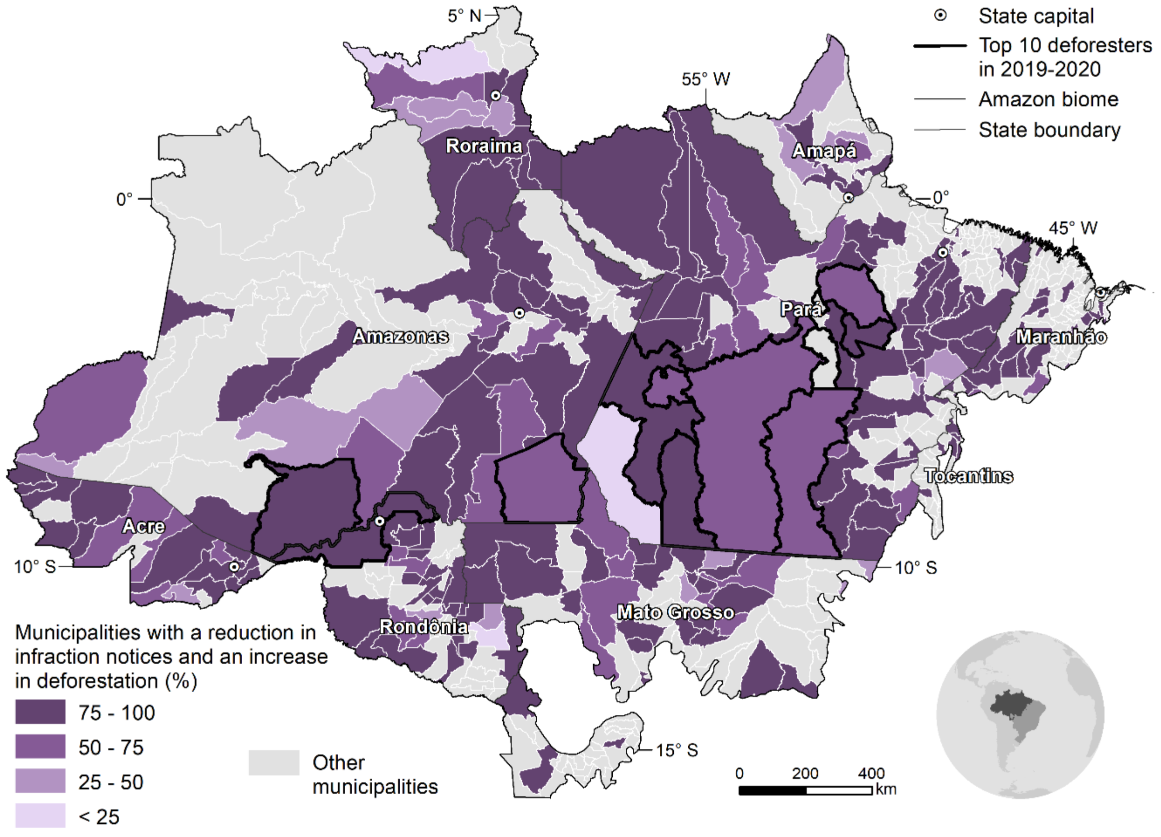

Fig. S8. Municipalities with reduced infraction notices issued and increased deforestation rates. Percentage of notification reduction in the 2014-2018 and 2019-2020 comparison. Sources: IBAMA (2021) and INPE (2021). Figure generated by ArcGIS Pro (https://www.esri.com/pt-br/arcgis/products/arcgis-pro/overview).

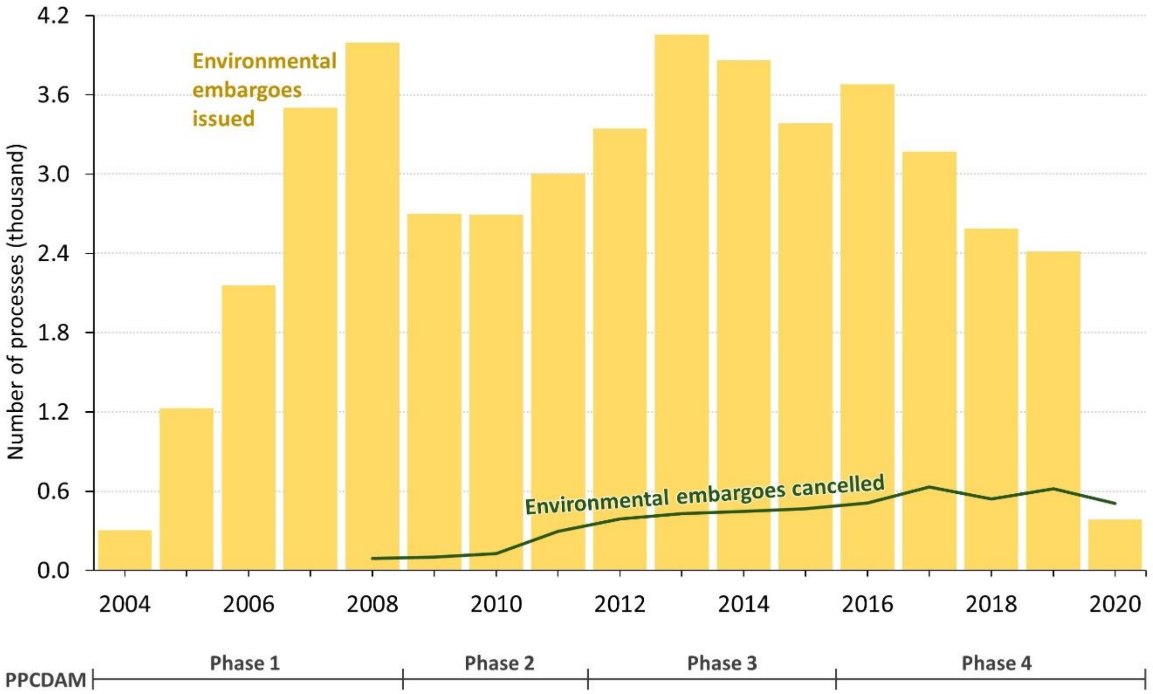

Fig. S9. Number of environmental embargoes issued and embargoes canceled throughout the PPCDAM.

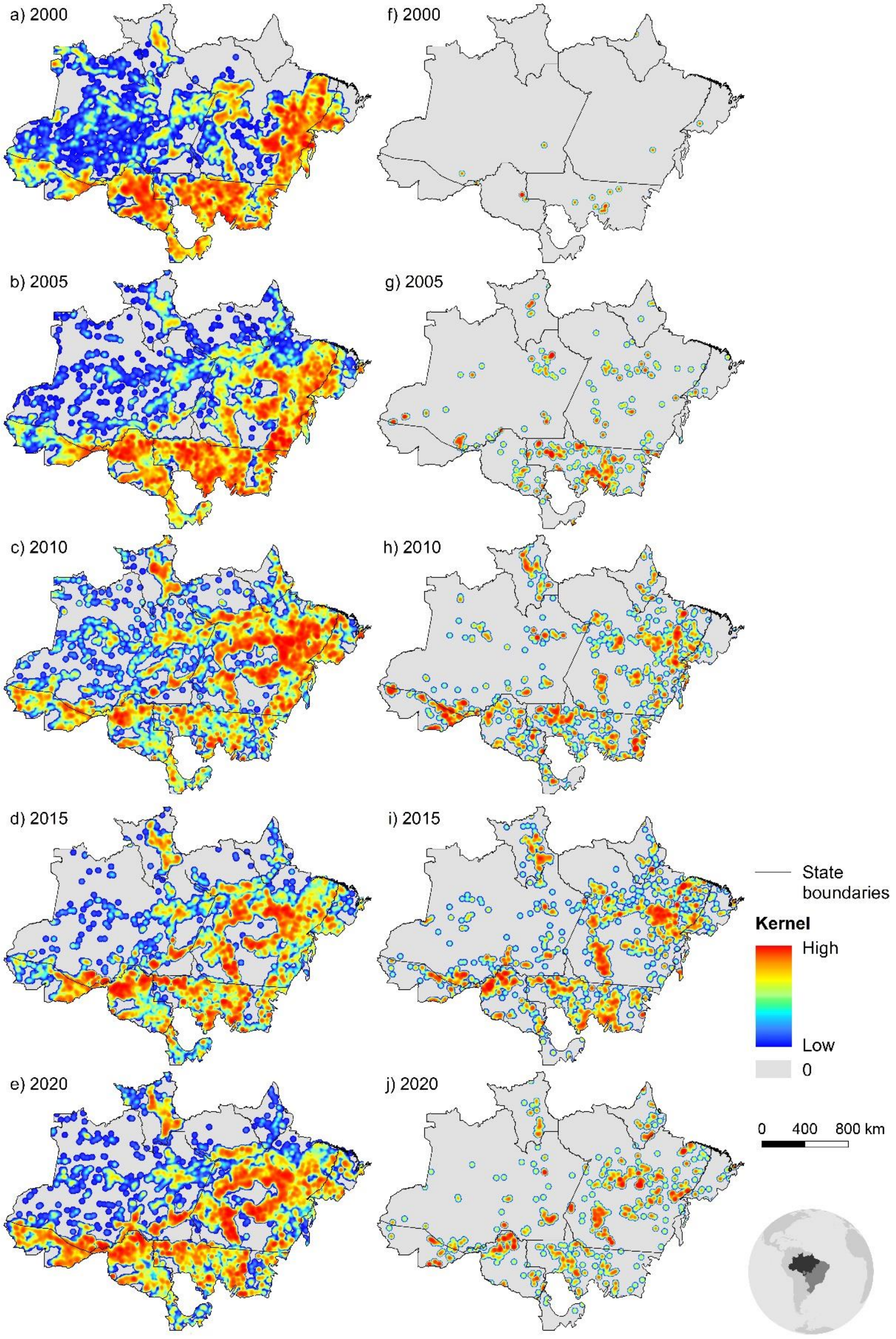

Fig. S10. Deforestation (a-e) and fines (f-j) hotspots in the Amazon. Sources: INPE (2021) and IBAMA (2021). Figure generated by ArcGIS Pro (https://www.esri.com/pt-br/arcgis/products/arcgis-pro/overview).

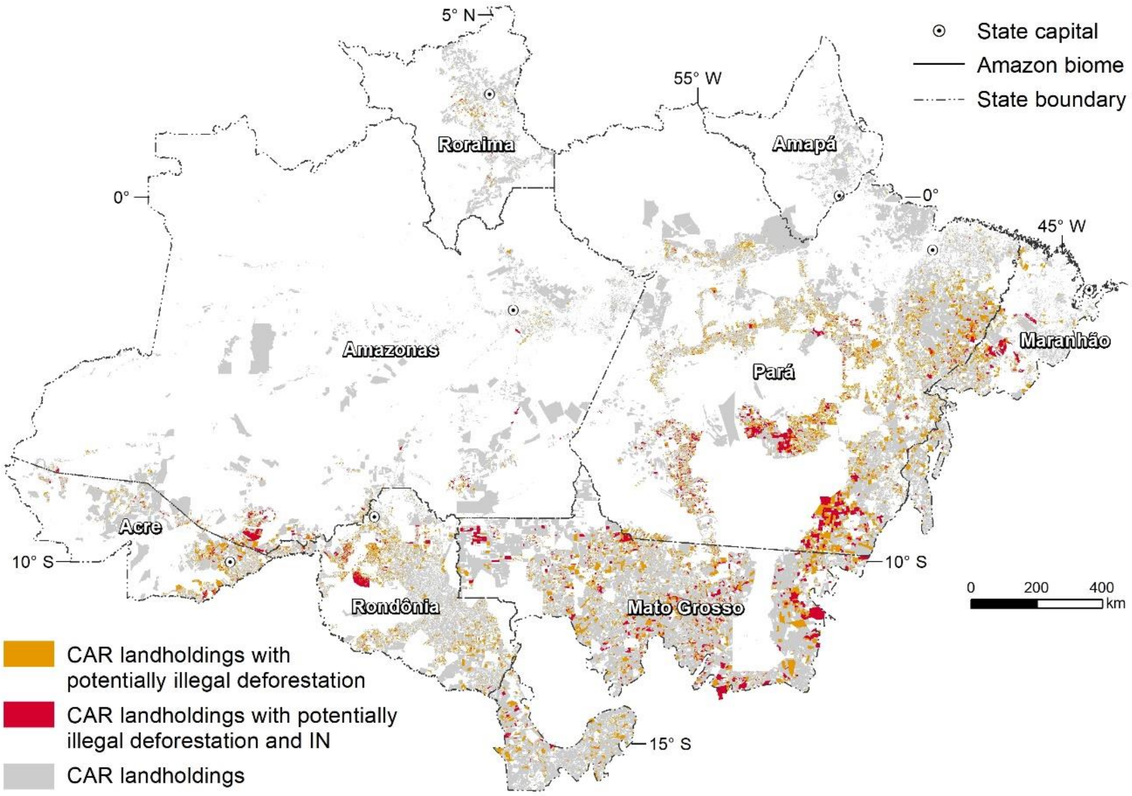

Fig. S11. Potentially illegal deforestation within CAR landholdings between 2009 and 2018 in the Amazon biome. CAR (national online environmental rural registry), IN (infraction notice). Source: IBAMA (2021).

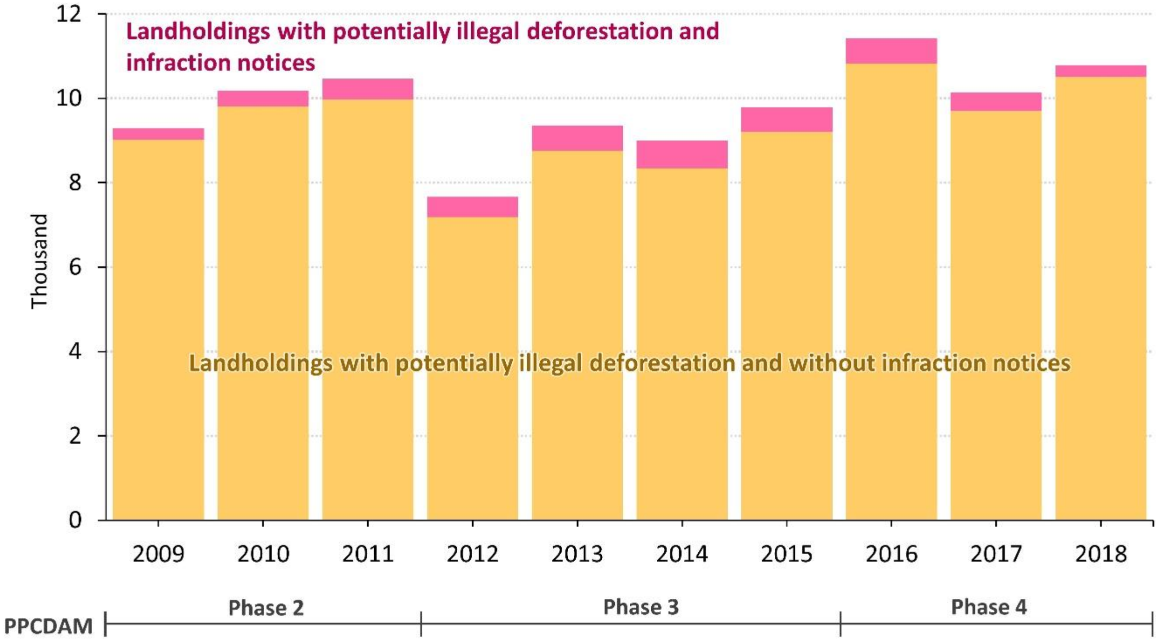

Fig. S12. Number of landholdings with potentially illegal deforestation with and without infraction notice in the Amazon biome. Source: IBAMA (2021).

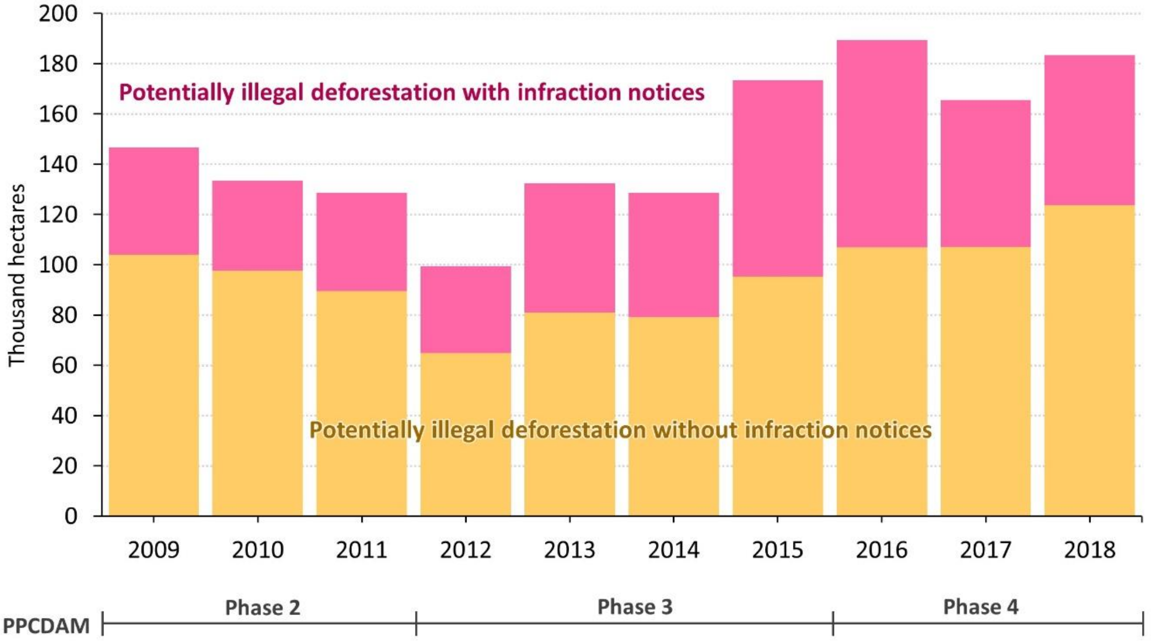

Fig. S13. Potentially illegal deforestation with and without infraction notice within landholdings in the Amazon biome. Source: IBAMA (2021).

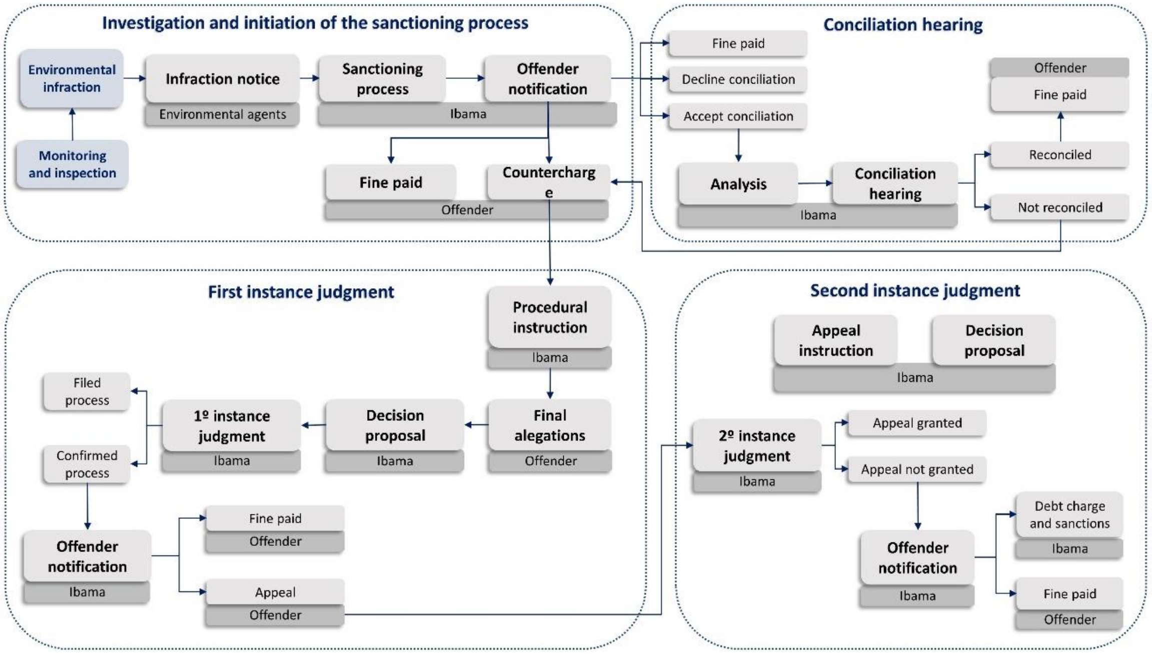

Fig S14: IBAMA´s sanctioning process and the additional phase of conciliation hearings before judgments.

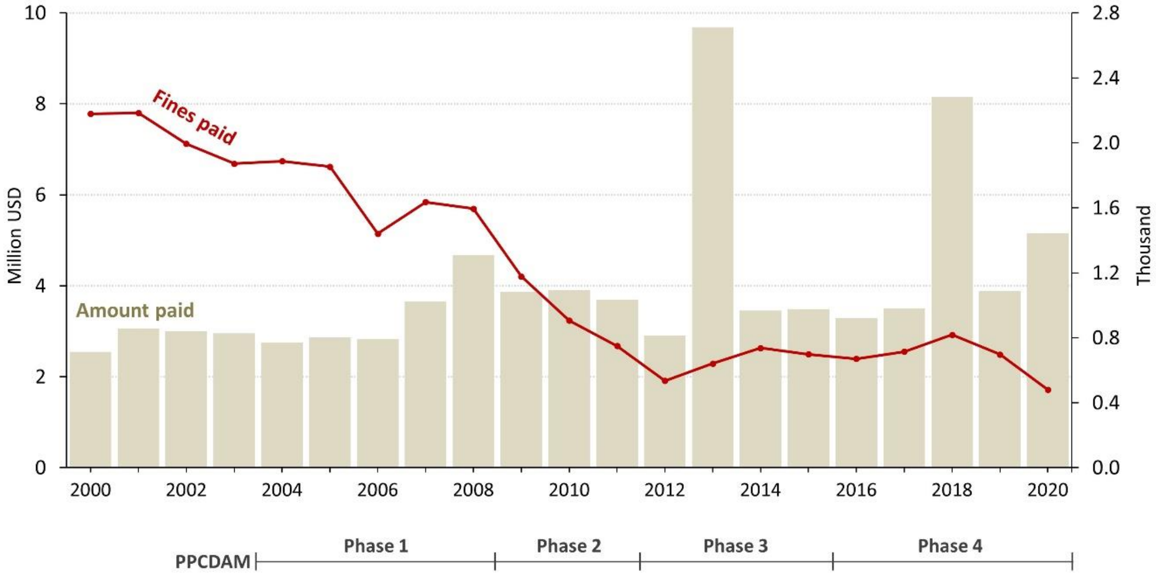

Fig. S15. Number of fines and amount paid throughout PPCDAM. Values were adjusted for inflation and converted to USD using a rate of R$ 5 per U$ 1 (BCB, 2021).

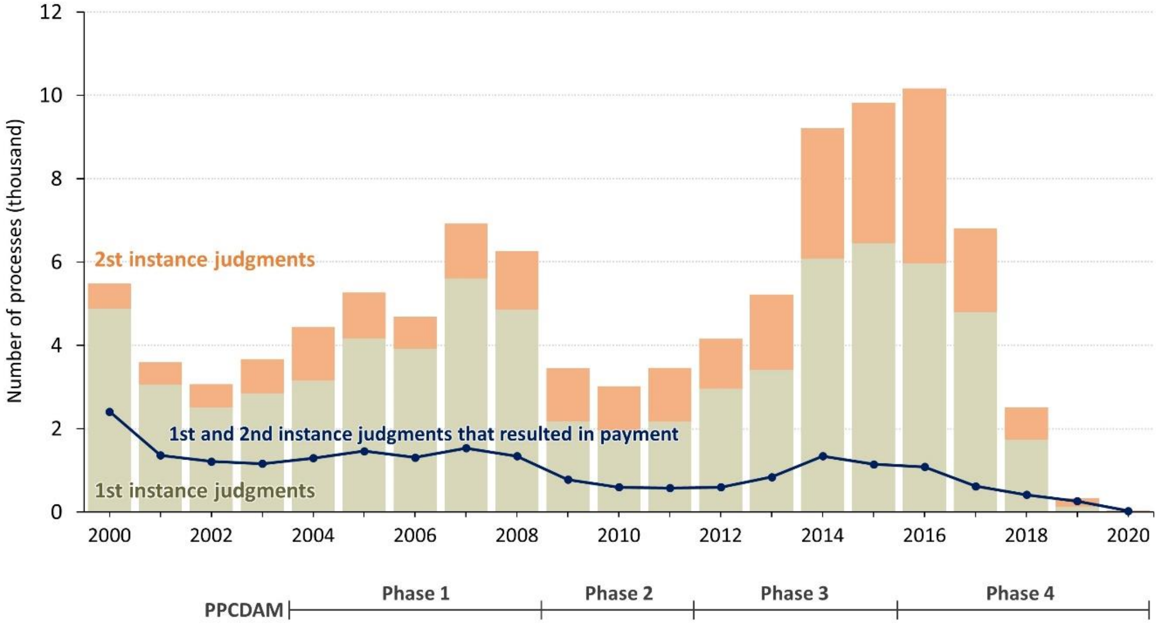

Fig. S16. Judgments of infraction notices and resulting fines paid in the Legal Amazon (IBAMA).

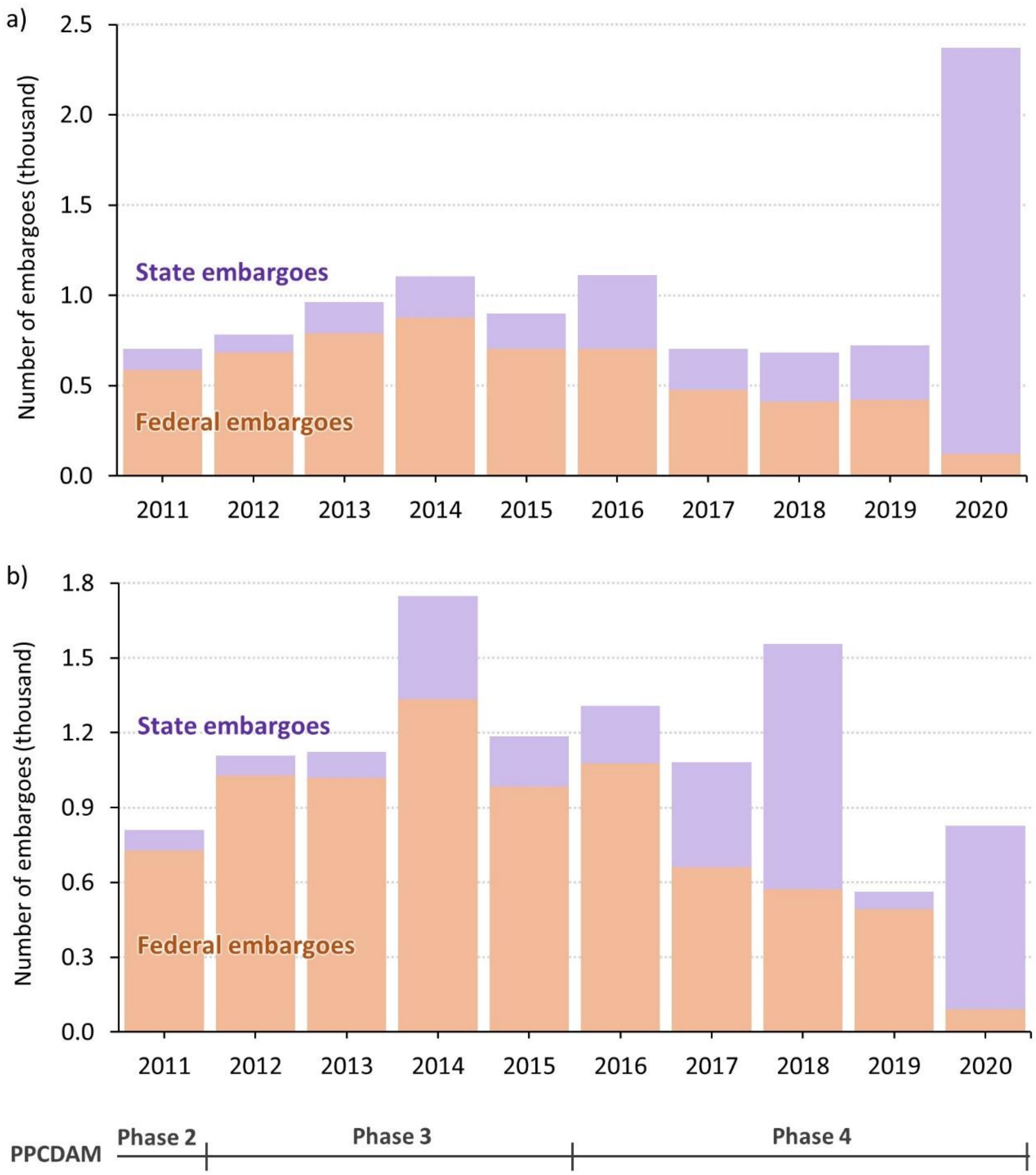

Fig. S17. Federal and state environmental embargoes in a) Mato Grosso and b) Pará. Pará and Mato Grosso accounted for roughly 53% of total deforestation in the Legal Amazon in the period 2011-2020 (INPE, 2022).

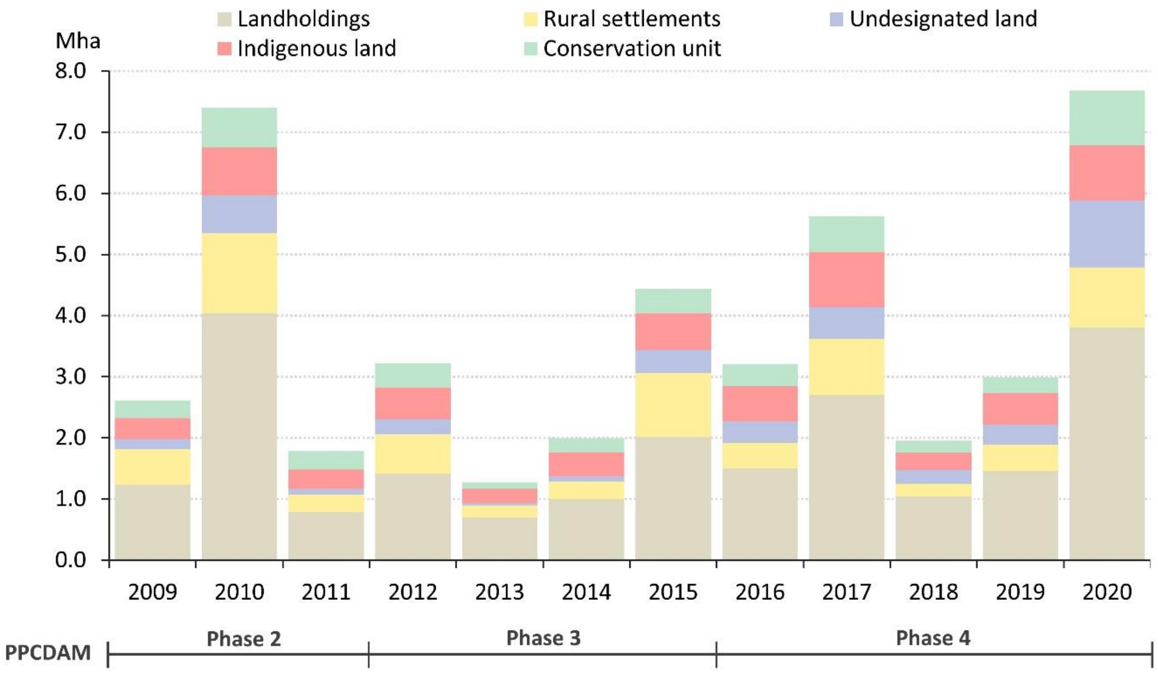

Fig. S18. Annual burned areas per land category from 2009 to 2020 in the Amazon biome (overlapped areas included). Source: NASA (2021).

Table S1. Legislative bills in order to downgrade, downsize or degazette federal CUs.

Status	Legislative bills
Denied	Writ of mandamus 25,347/2005
Denied	Draft legislative decree 149/2005
Denied	Writ of mandamus 25,346/2005
Denied	Law project 6,100/2005
Denied	Writ of mandamus 26,012/2006
Denied	Draft legislative decree 2,224/2006
Denied	Law project 6,479/2006
In progress	Law project 206/2007
Denied	Law project 4,083/2008
Denied	Draft legislative decree of the Chamber 1,148/2008
Denied	Law project 258/2009
Approved	Provisional measure 462/2009 (turned into law 12,058/2009)
Denied	Law project 6,927/2010
Approved	Provisional measure 472/2009 (turned into law 12,249/2010)
Approved	Law project 2,618/2011 (turned into ordinary law 13,090/015)
Denied	Provisional measure 542/2011
Approved	Provisional measure 558/2012 (turned into law 12,678/2012)
Denied	Draft legislative decree of the Chamber 914/2013
Denied	Law project 5,399/2013
Denied	Provisional measure 756/16
Approved	Provisional measure 758/2016 (turned into ordinary law 13,452/2017)
In progress	Law project 8,107/2017
In progress	Law project 6,024/2019

Table S2. Environmental fines and sanctions within landholdings in the states of Pará and Mato Grosso in 2020.

	Pará		Mato Grosso	
	**Number of landholdings**	**%**	**Number of landholdings**	**%**
Landholdings with deforestation in 2020*	10,360	4.0	3,487	2.3
Landholdings fined or embargoed for crimes against flora by the state agency	1,220	11.8	800	22.9
Landholdings fined or embargoed for crimes against flora by federal agencies	271	2.6	69	2.0
Landholdings fined or embargoed for crimes against flora by both state and federal agencies	1,353	13.1	841	24.1

**Deforestation >= 6.25 hectares.*

Table S3. Average deforestation in the Legal Amazon during the course of Green Brazil and Green Brazil 2 military operations.

	Historical deforestation (2015-2018)	Deforestation in 2019 (Verde Brasil)	Deforestation in 2020 (Verde Brasil 2)
From August to October	584 km^2	1,241 km^2	
From May to December	564 km^2		903 km^2

Table S4. Primary data sources and metadata.

Theme	Description	Source	Period covered
CAR	National environmental registry of rural properties (CAR)	IMAFLORA	2019
Conservation units	Conservation units	MMA	2021
Indigenous lands	Indigenous lands	FUNAI	2021
Land use	Land cover and land use	SEEG/Mapbiomas	2008
Hydrography	River streams and water bodies	ANA	2017
Deforested areas	Annual deforestation - PRODES project	INPE	2004-2020
Deforestation rates	Deforestation rates	INPE	2004-2021
Deforestation alerts	Deforestation alerts - DETER project	INPE	2004-2023
Expenses with environmental inspections	Net expenses of IBAMA and the Ministry of Defense	SIOP	2000-2020
Infraction notices	Infraction notices of environmental crimes	IBAMA	2000-2020
Embargos terms	Embargos terms of environmental crimes		2004-2020

Destruction terms	Destruction terms of environmental crimes		
Confiscation terms	Confiscation terms of environmental crimes		
Infraction notices	Infraction notices of environmental crimes	IBAMA	2019-2023
Embargos terms	Embargos terms of environmental crimes		
Destruction terms	Destruction terms of environmental crimes		
Confiscation terms	Confiscation terms of environmental crimes		
Embargos areas	Embargoed areas in the state of Mato Grosso	SEMA/MT	2011-2020
Infraction notices	Infraction notices in the state of Mato Grosso		
Embargos areas	Embargoed areas in the state of Pará	SEMAS/PA	2011-2020
Infraction notices	Infraction notices in the state of Pará		

The datasets used and/or analysed during this study are available by the corresponding author upon reasonable request.

SI References

Agência Nacional de Águas – ANA. Ottocoded hydrographic database 1:250.000. ftp://ftpana.ana.gov.br/BHO_2017/ (2017).

Banco Central do Brasil – BCB. Correção de valor por índices de preços. https://www3.bcb.gov.br/CALCIDADAO/publico/exibirFormCorrecaoValores.do?method=exibirFormCorrecaoValores&aba=1 (2021).

Fundação Nacional do Índio - FUNAI. Database. Terras indígenas. http://geoserver.funai.gov.br/geoserver/web/

Instituto Brasileiro do Meio Ambiente e dos Recursos Naturais Renováveis - IBAMA (2021). Consulta de Autuações Ambientais e Embargos. Accessed October 2022. Available in: <https://servicos.ibama.gov.br/ctf/publico/areasembargadas/ConsultaPublicaAreasEmbargadas.php>.

Instituto Brasileiro do Meio Ambiente e dos Recursos Naturais Renováveis – IBAMA. Portal Brasileiro de Dados Abertos. https://dados.gov.br/dataset?q=ibama (2023).

Instituto Nacional de Pesquisas Espaciais - INPE Projeto Prodes - Monitoramento de Desmatamento na Amazônia Legal. http://terrabrasilis.dpi.inpe.br/downloads/ (2021).

Instituto Nacional de Pesquisas Espaciais – INPE. DETER dados. http://www.dpi.inpe.br/obt/deter/dados/ (2023).

Instituto Nacional de Pesquisas Espaciais – INPE. Estimativa de desmatamento por corte raso na Amazônia Legal Brasileira. http://terrabrasilis.dpi.inpe.br/app/dashboard/deforestation/biomes/legal_amazon/rates (2022).

Ministério do Meio Ambiente – MMA. Unidades de conservação. Database. http://mapas.mma.gov.br/i3geo/datadownload.htm (2021).

National Aeronautics and Space Administration - NASA. NASA’s Level-1 and Atmosphere Archive and Distribution System - LAADS & Distributed Active Archive Center - DAAC. MODIS products. https://ladsweb.modaps.eosdis.nasa.gov/archive/allData/ (2021).

Secretaria de Estado de Meio Ambiente – SEMA/MT. Embargoes. Database. http://www.sema.mt.gov.br/transparencia/index.php/fiscalizacao-ambiental#332-arrecadacao-de-multas%3E (2021).

Secretaria de Estado de Meio Ambiente do estado do Mato Grosso – SEMA/MT. Autos de infração. Database: MVW_TIT_AUTUACAOPoint.shp. http://geo.sema.mt.gov.br/geoserver/Geoportal/ows?service=WFS&version=1.0.0&authkey=541085de-9a2e-454e-bdba-eb3d57a2f492&request=GetFeature&typeName=Geoportal:MVW_TIT_AUTUACAO&outputFormat=SHAPE-ZIP%3E (2021).

Secretaria de Estado de Meio Ambiente e Sustentabilidade do Pará – SEMAS/PA. Embargoes. Database: EMBARGOES_LDI_SEM_SOBREPOSICAO.shp. Available upon request (2021).

Secretaria de Estado de Meio Ambiente e Sustentabilidade do Pará – SEMAS/PA. Autos de infração. Database: auto_infracao.csv. Available by querying SEMAS´s database (2021).

Sistema de Estimativas de Emissões de Gases de Efeito Estufa do Observatório do Clima – SEEG. MapBiomas, Project - Collection 3.1 of Brazil's Annual Coverage and Land Use Map Series. https://mapbiomas.org/colecoes-mapbiomas-1?cama_set_language=pt-BR (2019).

Sistema Integrado de Planejamento e Orçamento do Governo Federal - SIOP. Acesso o SIOP. https://www.siop.planejamento.gov.br/modulo/login/index.html#/ (2022).
